# Clinician‐ and patient‐reported outcomes following the surgical treatment of single gingival recession defects: A systematic review

**DOI:** 10.1111/prd.12641

**Published:** 2025-07-22

**Authors:** Francesco Cairo, Emilio Couso‐Queiruga, Luigi Barbato, Cosimo Rupe, Sandra Stuhr, Leandro Chambrone, Gustavo Avila‐Ortiz

**Affiliations:** ^1^ Research Unit in Periodontology and Periodontal Medicine, Department of Clinical and Experimental Medicine University of Florence Florence Italy; ^2^ Department of Oral Surgery and Stomatology University of Bern School of Dental Medicine Bern Switzerland; ^3^ Department of Periodontics & Oral Medicine University of Michigan School of Dentistry Ann Arbor Michigan USA; ^4^ Evidence‐Based Hub, Centro de Investigação Interdisciplinar Egas Moniz (CiiEM) Egas Moniz School of Health & Science Almada Portugal; ^5^ Unit of Basic Oral Investigation Universidad El Bosque Bogota Colombia; ^6^ Department of Periodontics University of Pennsylvania Philadelphia Pennsylvania USA; ^7^ Department of Oral Medicine, Infection, and Immunity Harvard School of Dental Medicine Boston Massachusetts USA

**Keywords:** esthetics, gingival recession, patient‐reported outcomes, periodontium, plastic surgery

## Abstract

To analyze the effect of root coverage surgical therapy for the treatment of single gingival recession defects (GRD) in terms of clinician‐ and patient‐reported outcomes (CROs and PROs), with an emphasis on esthetic perception. The protocol of this PRISMA 2020‐compliant systematic review was registered in PROSPERO (CRD517050). Relevant articles reporting the outcomes of randomized controlled trials (RCTs) were identified through a literature search. After final article selection, according to specific eligibility criteria, data were extracted and categorized. Primary outcomes were clinician‐reported root coverage esthetic scores (RES) and patient‐reported esthetic perception and satisfaction using different assessment methods, such as standardized visual analog scales (VAS). Data were analyzed and the risk of bias in all included studies was assessed. Fifty‐eight articles pertaining to 50 different RCTs were selected. A total of 1820 subjects presenting 2219 single GRDs were treated. Key findings derived from the pooled estimates indicated that root coverage and gingival phenotype modification therapy positively influenced both RES and patient‐reported esthetic perception and satisfaction values. Compared to the use of a coronally advanced flap (CAF) alone (i.e., monolaminar technique), the use of a CAF in conjunction with a subepithelial connective tissue graft (i.e., bilaminar technique) had a positive impact on both RES and VAS values, whereas CAF in conjunction with soft tissue substitutes only had a beneficial effect on VAS values. Surgical approaches based on lateral flap displacement were associated with superior mean RES values compared to techniques involving coronal flap displacement. Meta‐regression analyses revealed a statistically significant positive association between mean root coverage and RES (i.e., the greater the percentage of root coverage, the higher the RES). Conversely, the association between patient‐reported esthetic perception and MRC was not statistically significant. In addition, it was observed that dentinal hypersensitivity can be substantially reduced with surgical root coverage therapy, regardless of the treatment modality. Surgical therapy for the correction of single GRDs had a positive effect on both clinician‐reported esthetic scores and patient‐reported esthetic perception and satisfaction. Bilaminar techniques are generally associated with superior results.

## 1 INTRODUCTION

Predictability of root coverage surgical interventions for the treatment of single gingival recession defects (GRDs) has been extensively demonstrated.^1^, ^2^, ^3^ In the majority of well‐conducted studies published in this field, the clinical efficacy of different approaches has been typically evaluated in terms of mean root coverage (mRC) and complete root coverage (CRC), as reported in previous systematic reviews.^1^, ^4^ Despite the introduction of new classifications, treatment guides, techniques, and materials,^5^, ^6^, ^7^, ^8^, ^9^ a systematic review published in 2022 concluded that the autogenous subepithelial connective tissue graft in conjunction with a coronally advanced flap can be considered the “gold standard” for the treatment of single GRs when mRC and CRC are the primary treatment outcomes.^10^

Nevertheless, as important as these clinician‐reported outcomes (CROs) are the success of root coverage interventions should not solely be determined by the level of coronal displacement of the gingival margin and changes in the soft tissue phenotype.^11^, ^12^ Other outcomes, such as patient‐perceived esthetics and overall patient satisfaction, should also be considered as part of a comprehensive set of therapeutic goals.^13^ In fact, clinician‐reported esthetic outcomes and patient‐reported outcomes (PROs) have been more frequently included in clinical studies published over the past decade.^5^, ^14^, ^15^, ^16^, ^17^, ^18^

While patient morbidity and satisfaction have been frequently evaluated with a visual analogue scale (VAS), several methods have been proposed to assess esthetic outcomes.^19^, ^20^, ^21^, ^22^, ^23^ Among them, the root coverage esthetic score (RES) introduced by Cairo and collaborators in 2009 has been shown to be a reliable tool for assessing the esthetic outcomes of surgical interventions aimed at achieving root coverage,^20^ not only for experts^24^ but also among operators with different experience levels.^25^

Previous systematic reviews have indicated that root coverage interventions contribute to improving esthetic perceptions by both patients and dentists^26^ and investigated the effect of different flap designs and graft materials for root coverage in terms of esthetics, patient satisfaction, and self‐reported morbidity.^27^ Nevertheless, considering the increase of available evidence in recent years on this topic, the conduction of a new systematic review, expanding the scope of clinical scenarios and surgical techniques applied, is justified.

The primary aim of this systematic review, which was conducted in the context of the European Federation of Periodontology (EFP) Focused Workshop on Esthetics and Patient‐Reported Outcomes in Periodontology and Implant Dentistry, was to analyze the effect of root coverage therapy for the treatment of single GRDs in terms of esthetic and PROs. The following focused question was addressed: “In patients with a clinical diagnosis of a single/localized GRD, what is the efficacy of root coverage surgical therapy in terms of clinician‐reported esthetic outcomes and patient‐reported outcomes?”

## 2 MATERIALS AND METHODS

The protocol of this review was registered in the International Prospective Register of Systematic Reviews (PROSPERO) with the identification code CRD42024517050. This review adheres to the guidelines of the Preferred Reporting Items for Systematic Reviews and Meta‐Analyses (PRISMA) 2020 statement.^28^ Based on a previous Cochrane systematic review,^1^ the following PICO framework was designed to address the review focused question:

**PICOS outline**

- Population: Adult patients with a clinical diagnosis of a single/localized GRD categorized as Miller class 1, 2, or 3, or RT 1 or 2.^29^, ^30^
- Intervention: Any type of surgical intervention for root coverage.
- Comparison: Any type of possible comparisons between surgical interventions for root coverage.
- Outcomes:
  ○ *Primary outcomes*:
    ▪ Clinician‐reported outcomes (CROs): Esthetic scores.
    ▪ Patient‐reported outcomes (PROs): Esthetic perception and satisfaction.
  ○ *Secondary outcomes*:
    ▪ Percentage of root coverage (%RC), percentage of complete root coverage (%CRC), changes in keratinized tissue width (ΔKTW), changes in gingival thickness (ΔGT), wound healing index (WHI), and other PROs (e.g., postoperative morbidity and complications, dentinal hypersensitivity, non‐esthetic concerns related to root coverage therapy).
- Study design: Randomized Controlled Trials (RCTs).

### 2.1 Criteria for inclusion

#### 2.1.1 Types of studies and participants

Only randomized controlled trials (RCTs) were considered eligible for inclusion if the participants met the following criteria:

- Surgical treatment of adult patients (aged ≥18 years) presenting single GRD.
- A follow‐up period of at least 6 months after the baseline surgical intervention.
- At least 10 participants per treatment arm at the final follow‐up examination.

#### 2.1.2 Types of interventions

Surgical interventions primarily aimed at root coverage and/or gingival phenotype modification (i.e., augmenting keratinized tissue width and/or thickness) were considered in this systematic review, namely:

- Free gingival graft (FGG).
- Laterally positioned flap (LPF).
- Coronally advanced flap (CAF).
- Tunnel flap technique (TUN).
- Subepithelial connective tissue graft (SCTG) in combination with LPF, CAF, or TUN.
- Soft tissue graft substitutes (i.e., allograft or xenograft matrices), and/or biologics (i.e., autologous blood‐products [ABPs], enamel matrix derivatives [EMD], or growth factors) in combination with LPF, CAF, or TUN.

Studies reporting on technical variations of the same type of intervention (e.g., flap without releasing incisions vs. flap with vertical releasing incisions, etc.) were included and categorized in the review.

### 2.2 Information sources and search strategy

Three electronic databases were searched, namely National Library of Medicine (MEDLINE/PubMed), Cochrane Central Register of Controlled Trials (CENTRAL) and EMBASE according to specific strategies (Table S1). The electronic search included articles published in the English language between January 1, 2000, and March 31, 2024. Additionally, a thorough hand search was performed by screening articles published in relevant scientific journals (i.e., *Journal of Clinical Periodontology*, *Journal of Periodontology*, *Journal of Periodontal Research*, *Clinical Oral Investigations*, and *The International Journal of Periodontics and Restorative Dentistry*), as well as recent systematic reviews on this topic,^26^, ^27^, ^31^ published between January 1st, 2000, and March 31st, 2024. Per consensus agreement with the EFP Focused Workshop Organizing Committee, the gray literature was not searched.

### 2.3 Selection process

Two reviewers (E.C.‐Q. and S.S.) independently performed the manual search and read the title and abstract of the entries obtained from the electronic search. Inter‐examiner calibration was achieved by open discussion and comparison following an independent assessment of the first 200 records. After completing the screening process, both reviewers read individually through the full‐text version of potentially eligible studies. Final article selection was dictated by the eligibility criteria (see Section 2.1). When disagreement regarding the inclusion or exclusion of a specific article occurred, both reviewers had an open discussion. If no agreement was achieved, other co‐authors (L.C. and G.A.‐O.) made the final decision. Cohen's kappa coefficient (*k*) was calculated to determine the degree of inter‐examiner agreement for final article selection.

### 2.4 Data extraction

Data extraction was preliminarily performed by two independent examiners (L.B. and C.R.). Examiners were calibrated by using a random selection of five articles to ensure consistency in the data extraction process and the terminology employed. Final data accuracy and consistency were independently verified by other co‐authors (E.C.‐Q., S.S., and L.C.). Any missing information that could contribute to this review was requested from the corresponding author(s) via email communication.

### 2.5 Methodological quality and risk‐of‐bias assessment

The assessment of methodological quality and risk of bias of each included RCT was performed in duplicate by two independent reviewers (E.C.‐Q. and S.S.) using version 2 of the Cochrane risk‐of‐bias tool for RCTs (RoB2).^32^ RoB2 individual domains assess the following: (1) bias arising from the randomization process; (2) bias due to deviations from intended interventions; (3) bias due to missing outcome data; (4) bias in the measurement of the outcome; and (5) bias in the selection of the reported result.

Based on this assessment, the risk of bias of each included RCT was rated as:

1. Low risk of bias (plausible bias unlikely to seriously alter the results) if all domains were at low risk of bias;
2. Unclear risk of bias (plausible bias that raises some doubt about the results) if ≥1 domains were at unclear risk of bias, but not to be at high risk of bias for any domain; or
3. High risk of bias (plausible bias that seriously weakens confidence in the results) if ≥1 domains were at high risk of bias, or the study is judged to have unclear risk of bias for multiple domains in a way that substantially lowers confidence in the result.

### 2.6 Data synthesis

Extracted data were organized into evidence tables. In addition to the outcomes of interest, supplemental data included year of publication and author(s), study design, setting(s) in which the study was conducted, sponsorship, initial number of participants, distribution per group, dropouts, and follow‐up period. Additionally, types of interventions, age, gender, smoking status, history of periodontitis, reason(s) for treatment, tooth and GRD type, recession (REC) depth and width, KTW, GT, and presence of non‐carious cervical lesions (NCCLs) were recorded.

Outcomes of interest were separated into short‐term (as evaluated 6–12 months following interventions), medium‐term (13–59 months), or long‐term (≥5 years).^1^, ^12^ When more than one short‐term (e.g., 6‐ and 12‐month results) and/or long‐term (e.g., 5‐ and 10‐year results) outcome was reported for the same variable, the longest follow‐up was included in the evidence tables.

### 2.7 Data analysis

Using the evidence tables, a descriptive summary was generated to assess the amount of data. Mixed‐effects multiple linear regression was undertaken using a statistical software package (Stata v.12.0, StataCorp, College Station, TX). Mean RES and mean VAS reported in individual treatment arms of selected studies(i.e., dependent variables) were employed to assess the association between gingival phenotype modification (“no” coded 0, “yes” coded 1), flap releasing incisions (“no” coded 0, “yes” coded 1), use of biologics (“no” coded 0, “yes” coded 1) and type of flap displacement (“coronal” coded 0, “lateral” coded 1), (i.e., independent variables) and clinician‐reported esthetic scores and patient‐reported esthetic perception and satisfaction values. Furthermore, regression models replacing “phenotype modification” with “type of graft material” (“no” coded 0, “SCTG” coded 1 and “autogenous graft substitutes” coded 2) were performed to detect differences between soft tissue grafting materials. Studies reporting mean VAS values using a 0–10 scale were adjusted to reflect 0–100 values, within the regression models. Additionally, meta‐regression analyses using mixed‐effect linear regression models and scatter plots with *R*‐squared values were carried out to assess the assumption that “clinician‐reported esthetic scores (specifically RES)” or “highest values of patient‐reported esthetic perception and satisfaction values (specifically VAS)” (i.e., dependent/outcome variable) could be predicted according to the “clinical outcomes (MRC)” (independent/explanatory variable) (e.g., whether the “highest values of VAS and RES” are associated with the “highest MRC” percentages). *p* Values < 0.05 were considered statistically significant.

## 3 RESULTS

### 3.1 Article selection

The initial database search yielded a total of 2003 entries, of which 448 were found in PubMed, 1253 in CENTRAL, and 302 in EMBASE. Four additional articles were identified through manual searching. Following removal of duplicates, 1189 entries remained. After title and abstract screening, 95 articles were selected for full‐text review. Thirty‐seven of these articles were excluded after full‐text review. The list of excluded articles and reasons for exclusion are displayed in Table S2.

Thus, 58 articles pertaining to 50 studies were finally selected.^15^, ^16^, ^19^, ^22^, ^23^, ^33^, ^34^, ^35^, ^36^, ^37^, ^38^, ^39^, ^40^, ^41^, ^42^, ^43^, ^44^, ^45^, ^46^, ^47^, ^48^, ^49^, ^50^, ^51^, ^52^, ^53^, ^54^, ^55^, ^56^, ^57^, ^58^, ^59^, ^60^, ^61^, ^62^, ^63^, ^64^, ^65^, ^66^, ^67^, ^68^, ^69^, ^70^, ^71^, ^72^, ^73^, ^74^, ^75^, ^76^, ^77^, ^78^, ^79^, ^80^, ^81^, ^82^, ^83^, ^84^, ^85^

**FIGURE 1:**
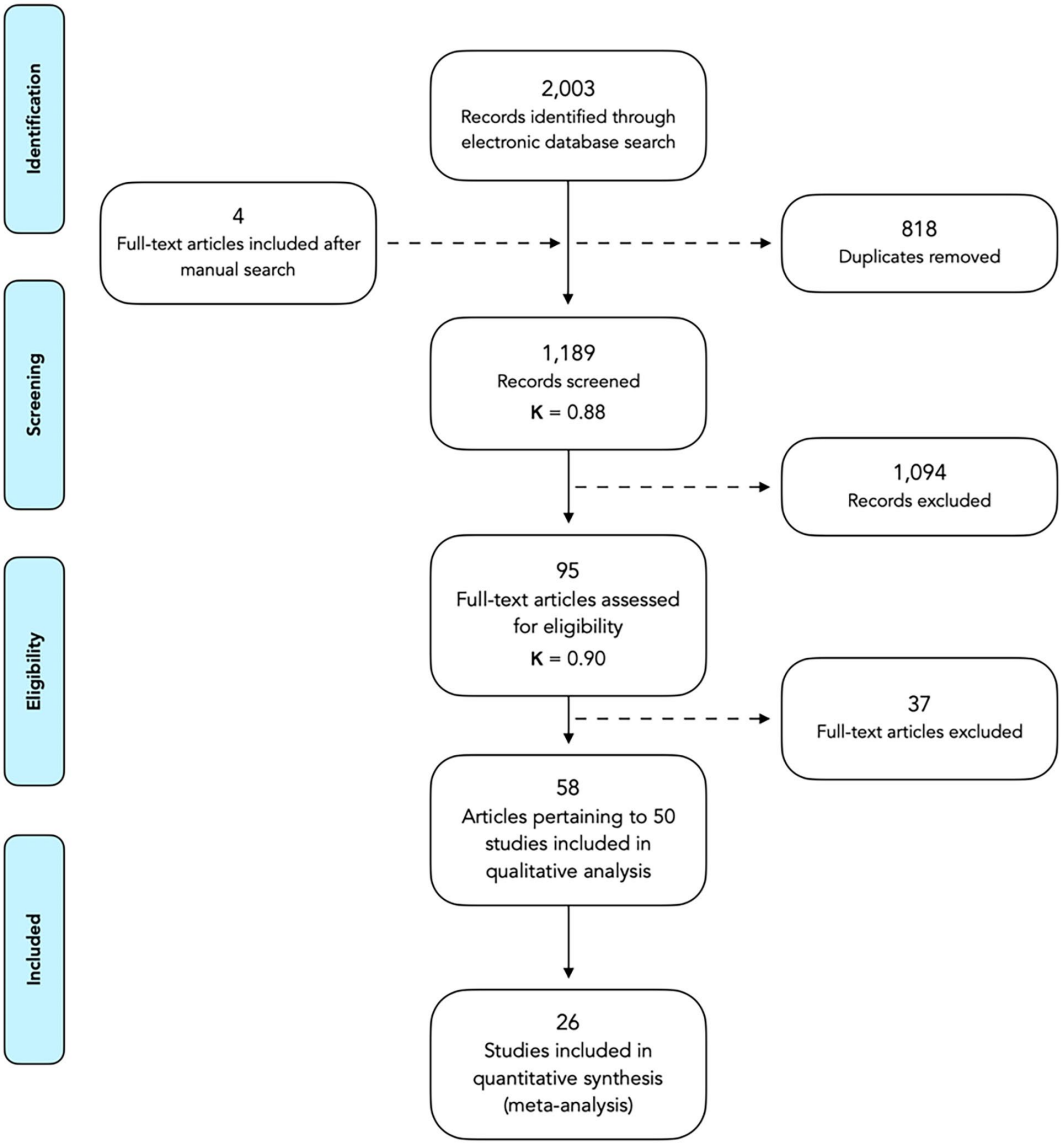
PRISMA flowchart depicting the article selection process.

A flowchart illustrating the article selection process is depicted in Figure 1. Inter‐examiner agreement kappa scores for title/abstract review and full‐text review were 0.88 and 0.90, respectively.

### 3.2 Risk‐of‐bias assessment

**FIGURE 2:**
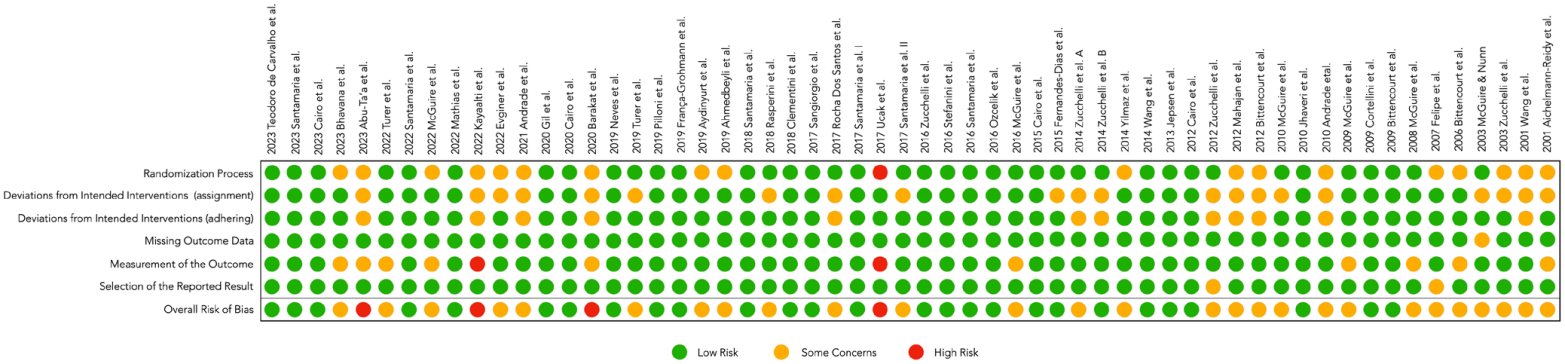
Risk‐of‐bias assessment.

As shown in Figure 2, of the 58 selected articles, 24 were categorized as having a low RoB, 4 exhibited a high RoB, and it was unclear in the remaining 30 papers.

### 3.3 Qualitative assessment

**FIGURE 3:**
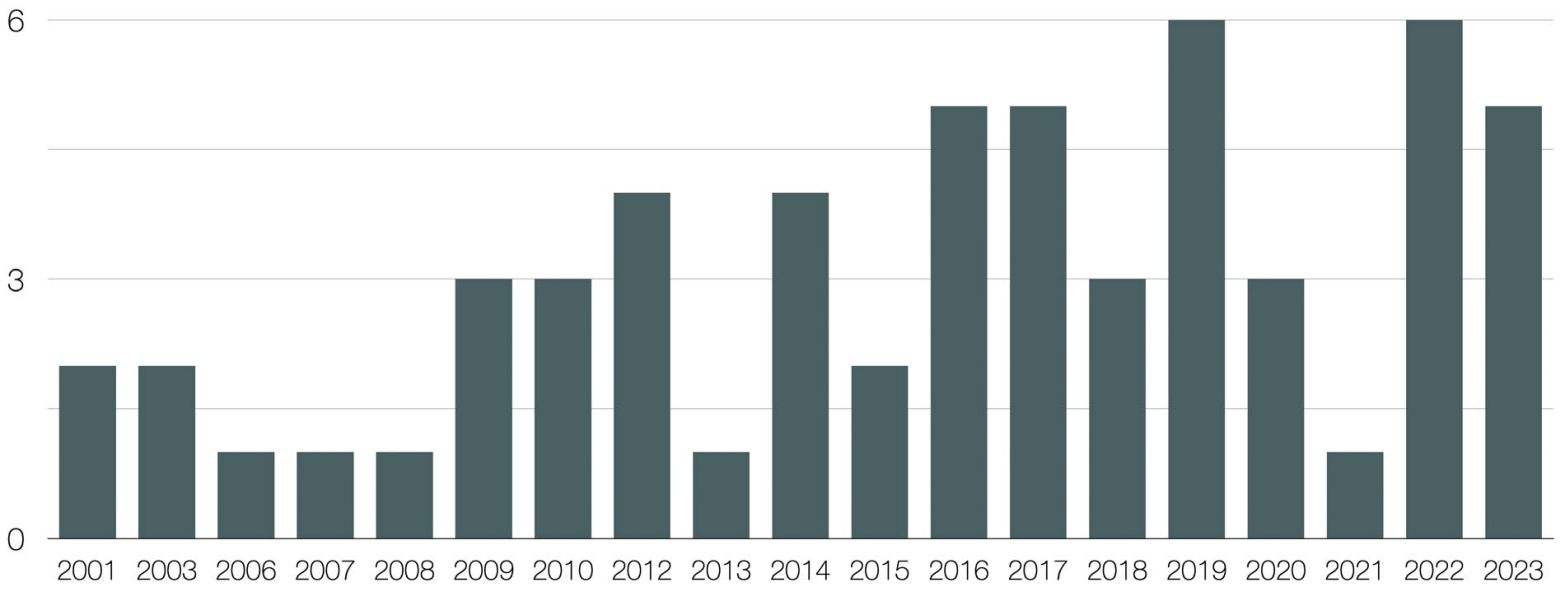
Bar graph depicting the distribution of selected articles per year of publication.

Of the 58 selected articles, 2 were published in 2001, 2 in 2003, 1 in 2006, 1 in 2007, 1 in 2008, 3 in 2009, 3 in 2010, 4 in 2012, 1 in 2013, 4 in 2014, 2 in 2015, 5 in 2016, 5 in 2017, 3 in 2018, 6 in 2019, 3 in 2020, 1 in 2021, 6 in 2022, and 5 in 2023, while no eligible articles published between January 1 and March 30, 2024, were identified (Figure 3). Only six RCTs were exclusively conducted in a private setting; the rest were either performed at universities or both at universities and private practices simultaneously. A total of 1820 patients presenting 2219 single GRD were treated through a variety of surgical interventions involving monolaminar and bilaminar techniques, either using autogenous grafts or substitutes. The concomitant use of biologic agents was reported in 10 articles, specifically EMD in 5 articles, ABPs in 4, and recombinant human platelet‐derived growth factor‐BB (rhPDGF‐BB) in one study. The follow‐up time after the baseline intervention ranged from 6 to 120 months. Only in two studies was the concomitant use of lasers reported.^63^, ^69^

Primary data on age and gender distribution, smoking status, history of periodontitis, reason(s) for treatment, tooth and GRD type, REC depth and width, KTW, GT, presence of non‐carious cervical lesions (NCCLs) and outcomes of interest is available in the data collection form in Appendix S2.

Notably, all interventions generally resulted in clinical improvements in terms of %RC, %CRC, RES values, and patient‐reported esthetic perception, overall satisfaction, and reduction of dentinal hypersensitivity.

#### 3.3.1 Relevant CROs

A total of 40 studies involved an esthetic evaluation by the investigators. Among them, 21 used the RES, while 3 employed a modified version of the RES (MRES),^41^, ^53^, ^62^ which accounts for the esthetic appearance of cervical restorations. Other methods of assessment were Likert scales and the QCE (Qualitative Cosmetic Evaluation).^50^, ^58^, ^65^ In particular, RES ranged from 6.7 to 9.1 for CAF (7 arms), and from 7.1 to 9.2 for LPF (5 arms). The CAF + SCTG was the most frequently investigated surgical approach (17 arms), which rendered RES values ranging from 6.65 to 9.40 (12 arms) and MRES values ranging from 7.52 to 8.1 (5 arms), while, for TUN + SCTG (3 arms) the RES ranged from 7.0 to 8.25. The use of soft tissue substitutes in conjunction with a CAF was also investigated. The application of a bilayer xenogeneic collagen matrix (XCM) was associated with RES that ranged from 6.9 to 7.85 (2 arms) and a MRES of 8.6 (1 arm). The use of platelet‐rich fibrin (PRF) was associated with RES that ranged from 6.9 to 8.3 (3 arms), while RES for acellular dermal matrix (ADM) and xenogeneic dermal matrix (XDM) was 8.1 (1 arm) and 6.6 (1 arm).

All the studies included in the present SR reported data on REC depth reduction and most of them on CRC. For details on the information reported in this section, see the data collection form in Appendix S2.

#### 3.3.2 Relevant PROs

Concerning PROs, 39 studies reported data on esthetics, 29 on postoperative morbidity, and 25 on dentinal hypersensitivity.

In 26 studies, patient‐reported esthetic satisfaction was measured using a 10‐point VAS. Studies on CAF (9 arms) and LPF (5 arms) reported VAS values ranging from 7.5 to 9.6 and from 7.7 to 8.9, respectively. Only one study reported the outcomes of a semilunar coronally positioned flap (mean VAS score was 8.2). The CAF + SCTG (19 arms) yielded VAS values that ranged from 7.38 to 9.5. One study assessed the patient‐reported esthetic satisfaction after TUN + SCTG, for a VAS value of 9.6. Studies on FGG (2 arms) reported a VAS value of 7.99 and 9.50. The use of soft tissue substitutes or biologics with a CAF rendered VAS values ranging from 8.58 to 9.4 for bilayer XCM (5 arms), 9.3 for XDM (1 arm), 8.67 for PRF (1 arm), and 8.63 for EMD (1 arm).

The use of VAS was the most common assessment method for postoperative morbidity (pain or discomfort) in the included studies. Although statistical analysis was not feasible, higher morbidity was reported for CAF + SCTG compared to CAF alone in single studies: 1.3 versus 0.8,^79^ 1.9 versus 1.0,^73^ 2.9 versus 2.4,^15^ 4.3 versus 2.75.^34^ Two studies assessed the effect of using a substitute with a CAF; Stefanini reported 2.04 for CAF versus 2.32 for CAF plus a bilayer XCM^16^ and Santamaria 2.6 for CAF versus 3.2 for CAF plus a bilayer XCM versus 3.8 for CAF + XDM.^39^ Unfortunately, none of the included studies comparing SCTG and soft tissue substitutes reported VAS values for postoperative morbidity.

Eighteen of the 25 publications reported dentinal hypersensitivity changes after therapy.^15^, ^39^, ^40^, ^41^, ^42^, ^43^, ^47^, ^50^, ^53^, ^54^, ^55^, ^57^, ^59^, ^60^, ^62^, ^65^, ^74^, ^79^ Notably, 15 of these publications were released since 2015, which illustrates the increasing interest in PROs from the research community in this field. All studies found a substantial reduction in dentinal hypersensitivity regardless of the treatment modality, with a few of them reporting a complete resolution after a follow‐up period of 6–108 months.^50^, ^54^, ^55^, ^57^, ^74^, ^79^ There did not seem to be a specific treatment modality associated with greater efficacy to predictably eliminate dentinal hypersensitivity.

Finally, due to data heterogeneity among very few studies, a meta‐analysis to explore the association between professional esthetic evaluation and patient perception was not possible. The observed tendency, however, seems to suggest a trend for having high‐RES values and high associated VAS values.

### 3.4 Quantitative assessment

Six sets of mixed‐effects linear regression, including data from 34 treatment arms for RES (models 1–3) and 33 for VAS (models 4–6), were performed to evaluate whether the use or type of soft tissue grafting material, use of biologics or flap design and management may have an influence on “clinician‐reported esthetic scores” and “patient‐reported esthetic perception and satisfaction values”.

Regression analyses were based on the following assumptions for the independent variable “soft tissue grafting material” (Tables 1 and 2).

**TABLE 1:** Mixed‐effects linear regression analysis evaluating factors influencing RES.

	Coef.	SE	*Z*	*p*‐Value	95% CI	
RES (Model 1)						
Phenotype modification (no/yes)	0.671	0.270	2.48	0.013*	0.140	1.201
Flap (releasing incisions—no/yes)	0.358	0.412	0.87	0.384	−0.449	1.167
Flap displacement (coronal/lateral)	0.833	0.338	2.47	0.014*	0.171	1.496
Biologics (no/yes)	0.498	0.379	1.31	0.189	−0.245	1.242
_cons	7.012	0.465	15.06	0.000	6.099	7.925
RES (Model 2)						
Graft (flap alone as the reference group)						
SCTG	0.927	0.275	3.37	0.001*	0.387	1.467
Soft tissue substitutes	0.165	0.334	0.50	0.620	−0.488	0.820
Flap (releasing incisions—no/yes)	0.577	0.395	1.46	0.144	−0.196	1.351
Flap displacement (coronal/lateral)	0.956	0.318	3.00	0.003*	0.331	1.580
Biologics (no/yes)	0.472	0.353	1.34	0.181	−0.219	1.164
_cons	6.755	0.447	15.11	0.000	5.879	7.632
RES (Model 3)						
Graft (SCTG as the reference group)						
Flap alone	−0.927	0.275	−3.37	0.001*	−1.467	−0.387
Soft tissue substitutes	−0.761	0.330	−2.30	0.021*	−1.409	−0.113
Flap (releasing incisions—no/yes)	0.577	0.395	1.46	0.144	−0.196	1.351
Flap displacement (coronal/lateral)	0.956	0.318	3.00	0.003*	0.331	1.580
Biologics (no/yes)	0.472	0.352	1.34	0.181	−0.219	1.164
_cons	7.683	0.352	21.81	0.000	6.992	8.374

Abbreviation: RES, root coverage esthetic score.

* Statistical significance.

**TABLE 2:** Mixed‐effects linear regression analysis evaluating factors influencing VAS.

	Coef.	SE	*Z*	*p*‐Value	95% CI	
VAS (Model 4)						
Phenotype modification (no/yes)	4.876	1.956	2.49	0.013*	1.041	8.712
Flap (releasing incisions—no/yes)	−5.577	4.989	−1.12	0.264	−15.356	4.201
Flap displacement (coronal/lateral)	−1.732	3.280	−0.53	0.598	−8.162	4.698
Biologics (no/yes)	3.941	2.462	1.60	0.109	−0.884	8.767
_cons	91.123	5.235	17.40	0.000	80.861	101.384
VAS (Model 5)						
Graft (flap alone as the reference group)						
SCTG	4.146	2.057	2.02	0.044*	0.114	8.178
Soft tissue substitutes	6.693	2.630	2.54	0.011*	1.537	11.849
Flap (releasing incisions—no/yes)	−6.215	4.953	−1.25	0.210	−15.923	3.492
Flap displacement (coronal/lateral)	−1.825	3.232	−0.56	0.572	−8.160	4.510
Biologics (no/yes)	3.631	2.444	1.49	0.137	−1.158	8.421
_cons	91.853	5.206	17.64	0.000	81.650	102.057
VAS (Model 6)						
Graft (SCTG as the reference group)						
Flap alone	−4.146	2.057	−2.02	0.044*	−8.178	−0.114
Soft tissue substitutes	2.546	2.511	1.01	0.311	−2.375	7.468
Flap (releasing incisions—no/yes)	−6.215	4.953	−1.25	0.210	−15.923	3.492
Flap displacement (coronal/lateral)	−1.825	3.232	−0.56	0.572	−8.160	4.510
Biologics (no/yes)	3.631	2.444	1.49	0.137	−1.158	8.421
_cons	96	4.782	20.07	0.000	86.626	

Abbreviation: VAS, visual analog scale.

* Statistical significance.

#### 3.4.1 Clinician‐reported esthetic scores using RES

##### Model 1

Whether the use of any kind of soft tissue graft for phenotype modification therapy (i.e., flap alone vs. soft tissue graft + flap), together with the other three independent variables, influenced RES: This model identified that procedures involving the use of a soft tissue graft or substitute (i.e., “gingival phenotype modification therapy”) with a LPF displayed superior RES outcomes.

##### Models 2 and 3

Whether the type of soft tissue grafting material (i.e., flap alone vs. flap + SCTG or allogeneic/xenogeneic substitute), together with the other three independent variables, influenced RES: These more refined models identified that: (a) Sites treated with SCTG displayed superior RES values compared to the use of a flap alone or a flap in conjunction with a soft tissue graft substitute; (b) sites treated with a LPF are associated with superior RES compared to the use of a CAF alone.

**FIGURE 4:**
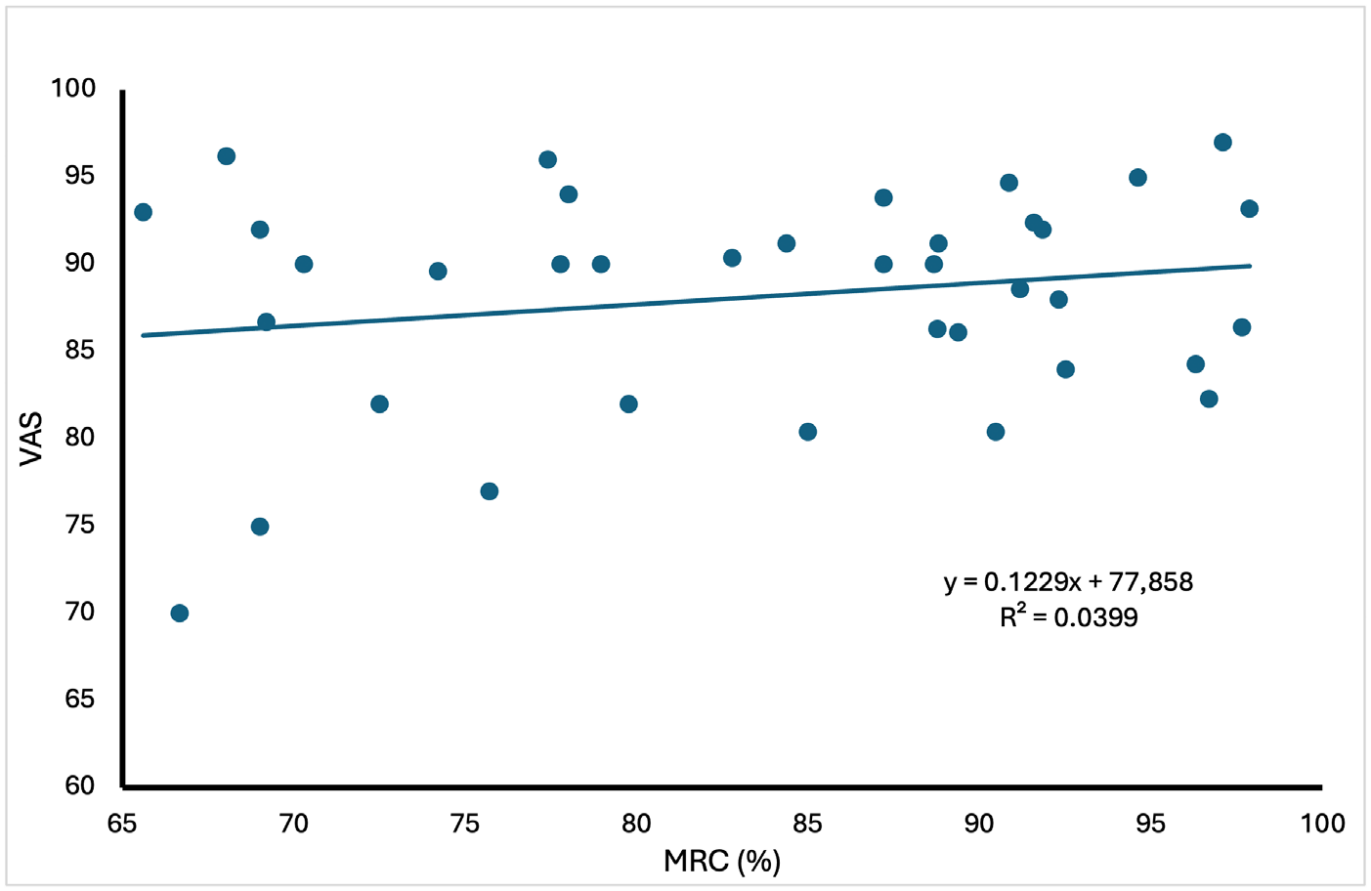
Scatter plot showing a linear neutral to positive correlation between VAS and MRC.

Moreover, meta‐regression (Coef: 0.03, SE: 0.01, *Z*: 2.91, *p* = 0.004, 95% CI 0.01–0.05) and scatter plot analyses revealed a statistically significant positive correlation between clinician‐reported esthetic scores and MRC (Table 1 and Figure 4).

#### 3.4.2 Patient‐reported esthetic perception and satisfaction values using VAS

##### Model 4

This model is similar to model 1 as it was constructed to assess whether phenotype modification therapy and the other three independent variables may have an influence on VAS values: In this model, soft tissue augmentation (i.e., bilaminar technique) was associated with a positive effect on VAS outcomes.

##### Models 5 and 6

Based on a similar approach as that applied in models 2 and 3, these regression analyses revealed that: (a) GRDs treated with SCTG or soft tissue substitutes displayed superior VAS values compared to the use of a flap alone; (b) the type of soft tissue grafting material, flap design, or management did not have an influence on VAS values.

**FIGURE 5:**
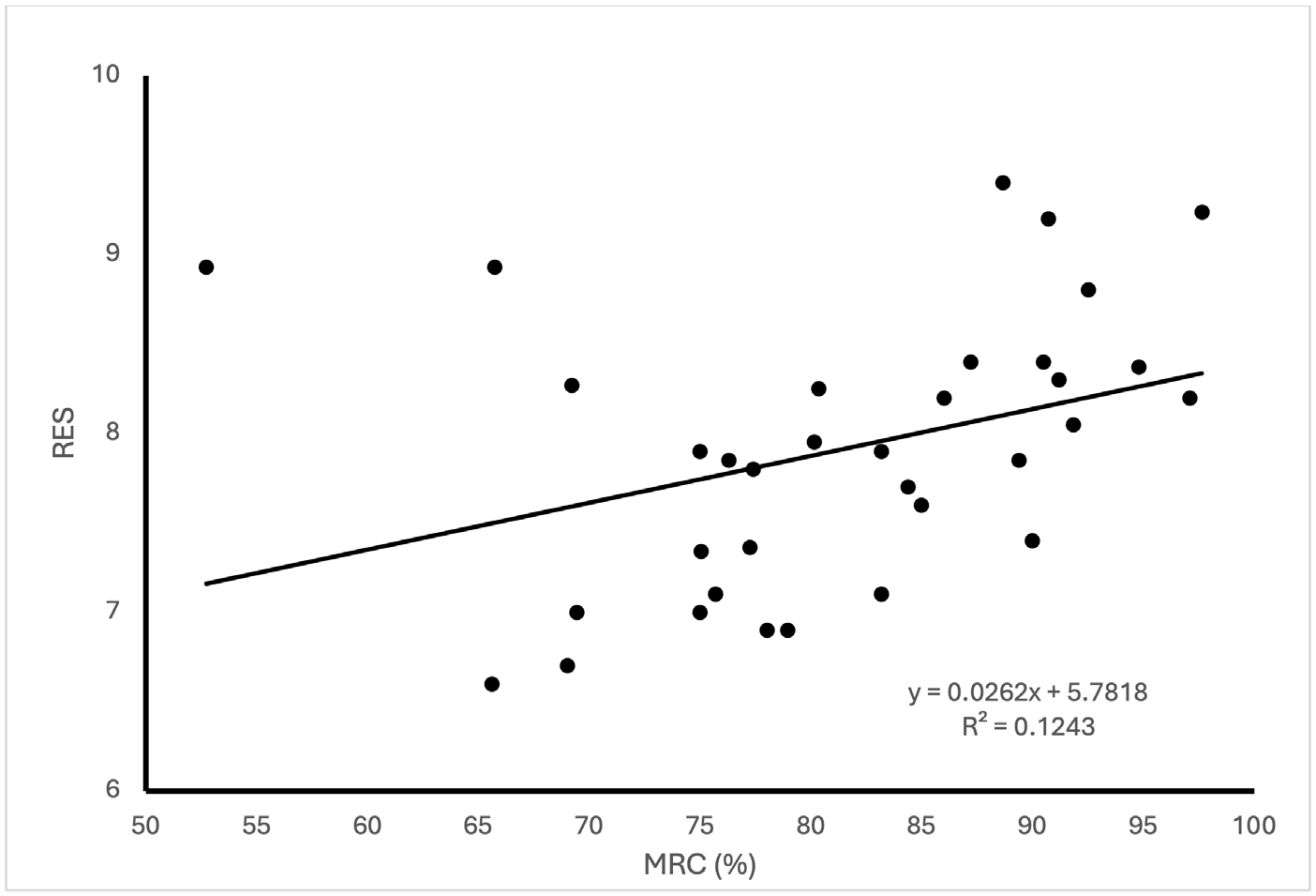
Scatter plot showing a linear positive correlation between RES and MRC.

Meta‐regression analyses (Coef: 0.12, SE: 0.09, *Z*: 1.24, *p* = 0.215, 95% CI −0.07 to 0.31) did not identify a statistically significant correlation between patient‐reported esthetic values and MRC (Table 2). In addition, a scatter plot showed a discrete linear neutral to positive correlation between VAS and MRC (Figure 5).

## 4 DISCUSSION

The present systematic review was primarily aimed at assessing the effect of root coverage and gingival phenotype modification therapy for the treatment of single GRDs in terms of CROs and PROs, with an emphasis on esthetics. A total of 58 RCTs including 1820 patients and 2219 GRDs were considered in the analysis.

### 4.1 Summary of main findings and agreements or disagreements with other studies or reviews

#### 4.1.1 Root coverage and gingival phenotype modification therapy typically result in favorable clinician‐ and patient‐reported esthetic outcomes

Outcomes from selected RCTs generally showed that surgical procedures aimed to achieve root coverage and GT gains, particularly bilaminar techniques, rendered favorable esthetic outcomes either assessed by the investigators or the patients. This finding is in alignment with existing evidence that suggests that the greater the percentage of root coverage, the higher the patient satisfaction.^15^, ^26^

When interpreting data regarding investigator‐assessed esthetic outcomes of root coverage therapy, it should be taken into consideration that the overall RES is largely sensitive to the amount of root coverage achieved, as it may account for up to 6 points out of a total of 10.^20^ For this reason, the meta‐regression analyses may have been affected by collinearity, which can cause problems in estimating the coefficients of the regression model. This aspect should be taken into consideration when interpreting their significance. Furthermore, RES values reported in some of the selected RCTs were heterogeneous. In particular, the RES ranged from 6.7 to 9.1 for CAF, 7.1 to 9.2 for LPF, 6.65 to 9.40 for CAF + SCTG, 7.0 to 8.25 for TUN + SCTG, and 6.9 to 7.85 for CAF + XCM. Nevertheless, a meta‐regression analysis (Coef: 0.03, SE: 0.01, *Z*: 2.91, *p* = 0.004, 95% CI 0.01–0.05) revealed a positive correlation between RES and MRC (Models 2 and 3).

Furthermore, our findings support a direct correlation between a greater percentage of root coverage and patient satisfaction, suggesting that favorable root coverage outcomes are noticed and valued by patients, which may be considered a “true endpoint” of periodontal plastic surgery. This notion is in line with the findings of a study on root coverage that used clinical photographs of GRDs from 100 patients.^86^ The investigators explored the perception of periodontists, general dentists, and patients regarding different results of root coverage therapy. It was observed that CRC was perceived as the most relevant outcome not only by professionals but also by patients, regardless of different educational backgrounds, age, and gender. Conversely, partial root coverage was considered satisfactory, especially when it was achieved in cases of deep REC in the presence of non‐dyschromic root surfaces.^86^

In summary, meta‐regression analyses revealed a statistically significant positive association between mean root coverage and RES (i.e., the greater the percentage of root coverage, the higher the RES). On the contrary, the association between patient‐reported esthetic perception and MRC, although positive, was not statistically significant.

#### 4.1.2 SCTG‐based bilaminar techniques are associated with superior clinician‐ and patient‐reported esthetic outcomes compared to other bilaminar modalities or monolaminar techniques

This SR confirmed that the use of CAF + SCTG was associated with superior RES outcomes than CAF combined with a substitute or a displaced flap alone (i.e., monolaminar technique). The superiority of CAF + SCTG for treating single GRDs has been extensively demonstrated in several RCTs published over the past two decades.^10^ It is generally acknowledged that SCTG acts as a biological filler that supports the primary flap, thus reducing postoperative flap shrinkage and promoting favorable root coverage outcome.^4^ This aspect may be critical in treating GRDs with loss of interdental attachment, where achieving adequate flap stability may be challenging. In fact, in an RCT on single RT2 defects that compared the effect of CAF + SCTG versus CAF alone, a higher probability of full root coverage (53% vs. 28%) and superior RES were observed in the CAF + SCTG group. This appeared to be particularly important in sites presenting with ≤3 mm of interproximal attachment loss.^73^

Furthermore, the benefits of SCTG‐based bilaminar techniques were also corroborated by long‐term observations, which showed superior gingival margin stability when an SCTG was used in comparison with other treatment modalities.^27^, ^87^ On the other hand, SCTG + CAF is a technique‐sensitive approach, as several variables, including graft size and thickness, may influence the final esthetic outcomes.^13^ This was demonstrated by Zucchelli et al., who conducted a RCT testing CAF in combination with a large and thick SCTG versus CAF with a SCTG presenting minimal thickness and dimensions similar to the dehiscence area. The authors reported similar root coverage outcomes in both groups, but superior esthetics and lower morbidity in the group that involved the use of a smaller and thinner SCTG.^84^

#### 4.1.3 The use of SCTG in combination with a CAF did not have a negative effect on gingival color and soft tissue texture

The results of our analysis on the effect of different surgical modalities of interest on individual RES variables revealed that the esthetic integration of SCTG was generally favorable (For details see Appendix S1). This finding contrasts with one of the main conclusions of a previous systematic review on this topic, which concluded that the use of a SCTG resulted in lower soft tissue texture and gingival color scores.^27^

This discrepancy may be explained by the expansion of the knowledge base and technical refinements that have been introduced in the field of periodontal plastic surgery over the past two decades, which have had a positive impact on clinician‐ and patient‐reported esthetic appraisals after treatment.^88^ Along with technical advancements, specific surgical instruments and magnification systems have been introduced, which allow for the execution of minimally traumatic surgical interventions, adequate flap and graft/substitute adaptation, and less surgical time, all of which contribute to promoting an uneventful healing period.

These considerations are important for an adequate interpretation of our findings that suggest that the modern CAF + SCTG does not seem to result in a subpar final esthetic appearance in terms of soft tissue texture (e.g., scar formation) and gingival color. Interestingly, these two variables are strongly correlated with improper graft positioning and excessive dimensions, which may lead to poor flap adaptation with graft exposure and significant soft tissue alterations.^20^ Key technical recommendations for the proper execution of a CAF + SCTG are the application of a graft with adequate dimensions that are adapted to the root dehiscence,^84^ along with proper flap design and management in terms of marginal thickness, tension, and position upon suturing.^88^, ^89^, ^90^ This notion is supported by the results of individual studies selected in this review: For CAF + SCTG procedures, in the studies published before 2020, RES scores >8/10 were only reported in 2 articles out of a total of 10. Conversely, 5 of 7 RCTs conducted after 2020 reported RES >8.

#### 4.1.4 Bilaminar techniques, either using a SCTG or a substitute, are associated with superior patient‐reported esthetic satisfaction compared to monolaminar techniques

Our analysis revealed that patient satisfaction is strongly related to root coverage outcomes, and bilaminar therapies able to increase soft tissue thickness are typically associated with superior patient‐reported esthetic satisfaction compared to monolaminar techniques. Interestingly, even though CAF + SCTG is generally acknowledged as the most effective therapeutic alternative to treat single GRDs,^10^ the use of soft tissue substitutes resulted in similar (non‐inferior) patient‐reported esthetic satisfaction.

This observation is in agreement with the findings from previous studies that reported high patient satisfaction and low morbidity after the use of graft substitutes in combination with a displaced flap for the treatment of single GRDs.^16^, ^41^, ^60^ These findings are also aligned with the growing interest in the development and testing of graft substitutes in periodontal plastic and reconstructive surgery to improve root coverage outcomes while reducing patient morbidity.^18^, ^39^, ^91^

#### 4.1.5 Impact of different root coverage therapy surgical modalities on patient‐reported morbidity and incidence of postoperative complications is uncertain

Patient‐related outcomes including perception of discomfort/pain are currently part of modern trials in periodontal plastic surgery. Unfortunately, data on pain and complications in studies selected for the conduction of the present systematic review were very heterogeneous, and a meta‐analysis was not feasible.

In most individual studies included in this review, surgical therapy for the correction of single GRDs seemed to be well tolerated by participants, with minimal painkiller consumption and moderate swelling limited to the first few days after surgery. However, outcomes of included studies suggest a tendency for higher postoperative pain for LPF and SCTG‐based procedures compared to CAF alone. Interestingly, some studies that involved the use of CAF + SCTG reported higher postoperative morbidity and use of painkillers, along with more surgical time, approximately 15 minutes, compared to other modalities.^15^, ^73^ Additionally, the presence of a second surgical site seems to be associated with a higher degree of complications, including pain and bleeding of the donor site, which emphasizes the importance of proper management and protection of the donor area to enhance the patient experience. However, these adverse events did not seem to influence either CROs or PROs in treated sites.

Data from individual RCTs seem to suggest that the use of soft tissue substitutes in conjunction with a displaced flap did not significantly increase patient morbidity compared with flap alone.^16^, ^60^ This finding supports the use of graft substitutes, such as collagen matrices, for root coverage therapy. Nevertheless, it is important to highlight that none of the included studies comparing SCTG and soft tissue substitutes reported VAS values for postoperative morbidity. Although it may be speculated that the lack of a second surgical site may be strongly related to lower morbidity, further clinical trials are needed for a quantitative evaluation of this potential advantage.

Notably, the incidence of severe postoperative complications (e.g., infection) in modern periodontal plastic surgery is low, in contrast with the high occurrence of such adverse events decades ago when the use of non‐resorbable barriers for root coverage was more extensive.

A meta‐analysis to explore the association between professional esthetic evaluation and patient perception was not possible due to few and heterogeneous data. Nevertheless, these studies reported a tendency for having high‐RES values and high associated VAS values. This observation seems to support previous outcomes for single recession treatment using CAF with or without collagen matrix, reporting a positive correlation between patient VAS and RES for both treatments.^16^

### 4.2 Limitations and potential biases in the review process

Regarding our quantitative analysis, it is important to note that only 52% of the selected studies were included in the different regression models, which should be considered when interpreting the pooled estimates. Moreover, it should be noted that both scatter plots displayed low *R*‐squared values (Figures 4 and 5), indicating that MRC may not precisely illustrate the performance of RES and VAS. This observation could be related to several factors: (1) The relatively small number of arms included in the analyses; (2) Individual perceptions of what a “successful esthetic treatment outcome” is; and (3) Data variability (e.g., differences in patient samples and study designs). On the other hand, *R*‐squared values are not indicative of biased regression models, as they simply indicate that the regression models accounted for a high degree of unexplainable variation. Also, it is important to remark that the statistically significant difference identified by the meta‐regression analysis denotes the positive influence of MRC on RES estimates. Finally, although postoperative morbidity and dentinal hypersensitivity were reported in 29 and 25 studies, respectively, a quantitative statistical analysis was not feasible due to methodological heterogeneity regarding the treatment arms and measures used to assess these outcomes.

## 5 CONCLUSIONS

Based on the findings of the present systematic review, it can be concluded that:

1. Favorable clinician‐reported esthetic scores and patient‐reported esthetic perception and satisfaction can be achieved with a variety of surgical modalities for the treatment of single GRDs.
2. Bilaminar techniques (i.e., connective tissue graft or substitute in conjunction with a Coronally Advanced Flap) were associated with superior RES values compared with monolaminar techniques (i.e., Coronally Advanced Flap alone).
3. Bilaminar techniques rendered superior patient‐reported esthetic outcomes compared with monolaminar techniques.
4. Different modalities of root coverage and gingival phenotype modification therapy seem to be generally well tolerated by patients, although the use of SCTG is typically associated with longer surgical time and postoperative morbidity compared to the application of substitutes or monolaminar techniques. Dentinal hypersensitivity can be substantially reduced with surgical root coverage therapy, although not completely resolved in a predictable manner, regardless of the treatment modality.
5. The relationship between clinician (RES) and patient‐reported satisfaction is still unclear and further researches are strongly encouraged to investigate this relevant issue.

### 5.1 Implications for future research and practice

Flap‐based monolaminar and bilaminar approaches may be effective for root coverage and gingival phenotype modification therapy in sites presenting single GRDs when esthetics is a priority. Bilaminar approaches involving the use of SCTG seem to be generally associated with superior CROs and PROs and therefore may be considered the gold standard. As an alternative, bilaminar surgical techniques using allogenic or xenogeneic soft tissue graft substitutes may be indicated when root coverage and gingival phenotype modification are the targets of treatment, whereas monolaminar approaches (i.e., LPF and CAF) may be employed when GT and/or KTW augmentation is not a goal of therapy. Future clinical studies on this topic should evaluate the effectiveness and efficacy of novel surgical modalities (e.g., involving the use of substitutes and/or biologics) in comparison to the gold standard (i.e., CAF + SCTG). These studies should consistently report a comprehensive panel of CROs and PROs with an emphasis on individual patient outcomes.

## AUTHOR CONTRIBUTIONS

G.A‐O. and F.C. conceived the idea and initial structure of the systematic review; L.C. assisted in the study design and performed the search; E.C.‐Q. and S.S. screened the initial entries and selected the articles; L.C. and G.A‐O. verified the final article selection according to protocol; L.B. and C.R. extracted the data; E.C.‐Q., S.S., and L.C. verified the validity and standardization of collected data; E.C.‐Q. and S.S. assessed the risk of bias; L.C. contributed to refining the structure of the manuscript and data analysis; G.A‐O., L.C., and F.C. led the writing of the manuscript; E.C.‐Q., L.B., C.R., and S.S. critically revised the final manuscript.

## CONFLICT OF INTEREST STATEMENT

The authors have no conflicts of interest to report pertaining to the conduction of this systematic review.

## Supporting information


Appendix S1

Appendix S2

Table S1

Table S2

## Data Availability

The data that support the findings of this study are available from the corresponding authors upon reasonable request.
