# Supplementary material for: Clinician‐ and patient‐reported outcomes following the surgical treatment of single gingival recession defects: A systematic review
Source: Periodontol 2000. 2025 Jul 22;99(1):7–20. doi: 10.1111/prd.12641 (PMC13428094; doi:10.1111/prd.12641)
Supplement: Supplementary file 1 — Appendix S1 [file PRD-99-7-s002.docx]

**Appendix S1.** Mixed-effects linear regression analyses assessing the relationship between individual components of the RES (i.e., complete root coverage [CRC] as part of gingival margin [GM] position, marginal tissue contour [MTC], soft tissue texture [STT], mucogingival junction alignment [MGJ], gingival color [GC]) and technical variables (i.e., flap alone [monolaminar approach], type of grafting material [bilaminar approach] and flap displacement).

| CRC (RES) | Coef. | SE | Z | P-value | 95% CI | |
| --- | --- | --- | --- | --- | --- | --- |
| Graft (Flap alone as the reference group)  SCTG  Soft tissue substitutes | 0.556  0.916 | 0.290  0.403 | 1.92  2.27 | 0.055  0.023* | -0.012  0.126 | 1.124  1.706 |
| Flap displacement (coronal/lateral) | 0.419 | 0.329 | 1.27 | 0.203 | -0.225 | 1.064 |
| _cons | 4.013 | 0.217 | 18.43 | 0.000 | 3.587 | 4.440 |

| CRC (RES) | Coef. | SE | Z | P-value | 95% CI | | |
| --- | --- | --- | --- | --- | --- | --- | --- |
| Graft (SCTG as the reference group)  Flap alone  Soft tissue substitutes | -0.556  0.360 | 0.290  0.438 | -1.92  0.82 | 0.055  0.412 | -1.124  -0.499 | 0.012  1.220 | |
| Flap displacement (coronal/lateral) | | 0.419 | 0.329 | 1.27 | 0.203 | -0.225 | 1.064 |
| _cons | 4.57 | 0.191 | 23.83 | 0.000 | 4.194 | 4.945 | |

RES – root coverage esthetic score; * signifies statistical significance

| MTC (RES) | Coef. | SE | Z | P-value | 95% CI | |
| --- | --- | --- | --- | --- | --- | --- |
| Graft (Flap alone as the reference group)  SCTG  Soft tissue substitutes | 0.184  0.110 | 0.069  0.096 | 2.65  1.14 | 0.008*  0.253 | 0.048  -0.079 | 0.321  0.300 |
| Flap displacement (coronal/lateral) | 0.095 | 0.079 | 1.21 | 0.225 | -0.059 | 0.250 |
| _cons | 0.716 | 0.052 | 13.71 | 0.000 | 0.614 | 0.819 |

| MTC (RES) | Coef. | SE | Z | P-value | 95% CI | | |
| --- | --- | --- | --- | --- | --- | --- | --- |
| Graft (SCTG as the reference group)  Flap alone  Soft tissue substitutes | -0.184  -0.74 | 0.069  0.105 | -2.65  -0.71 | 0.008*  0.480 | -0.321  -0.280 | -0.048  0.132 | |
| Flap displacement (coronal/lateral) | | 0.095 | 0.079 | 1.21 | 0.225 | -0.059 | 0.250 |
| _cons | 0.901 | 0.046 | 19.59 | 0.000 | 0.811 | 0.991 | |

RES – root coverage esthetic score; * signifies statistical significance

| STT (RES) | Coef. | SE | Z | P-value | 95% CI | |
| --- | --- | --- | --- | --- | --- | --- |
| Graft (Flap alone as the reference group)  SCTG  Soft tissue substitutes | 0.027  0.230 | 0.094  0.131 | 0.29  1.75 | 0.769  0.081 | -0.158  -0.028 | 0.213  0.488 |
| Flap displacement (coronal/lateral) | 0.100 | 0.107 | -0.93 | 0.353 | -0.310 | 0.110 |
| _cons | 0.605 | 0.071 | 8.50 | 0.000 | 0.465 | 0.744 |

RES – root coverage esthetic score; * signifies statistical significance

| MGJ (RES) | Coef. | SE | Z | P-value | 95% CI | |
| --- | --- | --- | --- | --- | --- | --- |
| Graft (Flap alone as the reference group)  SCTG  Soft tissue substitutes | 0.041  0.106 | 0.066  0.092 | 0.63  1.16 | 0.532  0.248 | -0.088  -0.074 | 0.172  0.288 |
| Flap displacement (coronal/lateral) | -0.084 | 0.075 | -1.12 | 0.262 | -0.232 | 0.063 |
| _cons | 0.865 | 0.049 | 17.33 | 0.000 | 0.767 | 0.963 |

RES – root coverage esthetic score; * signifies statistical significance

| GC (RES) | Coef. | SE | Z | P-value | 95% CI | |
| --- | --- | --- | --- | --- | --- | --- |
| Graft (Flap alone as the reference group)  SCTG  Soft tissue substitutes | -0.014  -0.018 | 0.028  0.039 | -0.51  -0.46 | 0.607  0.643 | -0.070  -0.096 | 0.041  0.059 |
| Flap displacement (coronal/lateral) | -0.082 | 0.032 | -2.54 | 0.011* | -0.146 | -0.018 |
| _cons | 0.954 | 0.021 | 44.43 | 0.000 | 0.912 | 0.996 |

RES – root coverage esthetic score; * signifies statistical significance
