# Supplementary material for: Clinician‐ and patient‐reported outcomes following the surgical treatment of single gingival recession defects: A systematic review
Source: Periodontol 2000. 2025 Jul 22;99(1):7–20. doi: 10.1111/prd.12641 (PMC13428094; doi:10.1111/prd.12641)
Supplement: Supplementary file 2 — Appendix S2 [file PRD-99-7-s004.pdf]

Study code	**Publication(s)** Year and Author(s)
1	2001 Aichelmann-Reidy et al.
2	2001 Wang et al.

3	2003 Zucchelli et al.
4	2003 McGuire & Nunn

5	2006 Bittencourt et al.
5 F/UP	2009 Bittencourt et al. (Follow-up of 2006 Bittencourt et al.)

6	2007 Felipe et al.
7	2008 McGuire et al.
8	2009 Cortellini et al.

8 F/UP	2018 Rasperini at al.<br>(subsample follow-up study form Cortellini 2009)
9	2009 McGuire et al.
10	2010 Andrade et al.

11	2010 Jhaveri et al.
12	2010 McGuire et al.
12 F/UP	2016 McGuire et al.<br>(Follow-up of 2010 MCGuire et al.)
13	2012 Bittencourt et al.
14	2012 Mahajan et al.

15	2012 Zucchelli et al.
16	2012 Cairo et al.

16 F/UP 1	2015 Cairo et al.<br>(follow-up of 2012 Cairo et al.)
16 F/UP 2	2023 Cairo et al.<br>(follow-up of 2012 Cairo et al.)
17	2014 Wang et al.
18	2014 Yilmaz et al.

19	2014 Zucchelli et al. A
20	2014 Zucchelli et al. B
21	2015 Fernandes-Dias et al.

21 F/UP	2017 Santamaria et al. (follow-up of 2015 Fernandes-Dias et al.)
22	2016 Ozcelik et al.
23	2016 Santamaria et al.

24	2013 Jepsen et al.<br>2016 Stefanini et al.
25	2016 Zucchelli et al.

26	2017 Rocha Dos Santos et al.<br>2017 Sangiorgio et al.
27	2017 Ucak et al.
28	2017 Santamaria et al.

28 F/UP	2019 da Silva Neves et al.<br>(follow-up of 2017 Santamaria et al)
29	2018 Clementini et al.
30	2018 Santamaria et al.
31	2019 Ahmedbeyli et al.
32	2019 Aydinyurt et al.

33	2019 França-Grohmann et al.
34	2019 Pilloni et al.
35	2019 Turer et al.

36	2020 Barakat et al.
37	2020 Cairo et al.
38	2020 Gil et al.

39	2021 Andrade et al.
40	2022 Evginer et al.

41	2022 Kayaalti et al.
42	2022 Mathias-Santamaria et al.

43	2022 McGuire et al.
44	2022 Santamaria et al.

45	2022 Turer et al.
46	2023 AbuTa'a et al.
47	2023 Bhavana et al.
48	2023 Santamaria et al.

49	2023 Teodoro de Carvalho et al.

General Study Ch

**Study design** Parallel-arms (PA) or Split-mouth (SM)	**Setting**
SM	University
SM	University

SM	University
SM	Private

SM	University
SM	University

SM	University
SM	Private
PA	Mixed

PA	University
SM	Private
PA	University

SM	University
SM	Private
SM	Private
SM	University
PA	University

PA	University
PA	University

PA	University
PA	University
PA	University - Multicenter
PA	University

PA	University
PA	University
PA	University

PA	University
PA	University
PA	University

SM	University - Multicenter
PA	University

PA	University
PA	University
PA	University

PA	University
PA	University
PA	University
PA	University
SM	University

PA	University
PA	University
PA	University

SM	University
PA	University
PA	University

P/SM (mixed)<br>Most Patients Received two treatments, although a few received up to four treatments, following the randomization schedule	University
PA	University

SM	University
PA	University

SM	Private - Multicenter
PA	University

PA	University
SM	University
PA	University
PA	University

SM	University

naracteristics and Part

Sponsor
LifeCell Corp., and the Louisiana Periodontics Support Fund
Sulzer Calcitek Inc., Carlsbad, California

N/R
Biora AB, Malmoe (Sweden)

Research Funding Agency
from Saõ Paulo State

Research Funding Agency
from Saõ Paulo State

State of São Paulo Research foundation
Organogenesis - Canton (USA)
Accademia Toscana di Ricerca Odontostomatologica (ATRO), and the European Research Group on Periodontology, ERGOPerio

No financial support
Osteohealth, a division of Luitpold Pharmaceuticals, Shirley, New York
State of São Paulo Research foundation and the National Counsel of Technological and Scientific Development, Brasilia

The International Clinical Dental Research Organization (India)
Partly private<br>Geistlich Pharma
Partly private<br>Geistlich Pharma
Research Funding Agency of Bahia State, Brazil
N/R

No financial support
No financial support

No financial support
No financial support
Zimmer Dental
No financial support

No financial support
No financial support
Research Funding Agency from São Paulo State (FAPESP), Brazil grant # 2012/14595-3

Research Funding Agency from São Paulo State (FAPESP), Brazil grant # 2012/14595-3
No financial support
Research Foundation of the State of Sao (Grant #2009/17194-7) and CNPq, National Council for Scientific and Technological Development (Grant #485408/2011-2).

Geistlich Pharma AG
N/R

São Paulo State research funding agency São Paulo Research Foundation (FAPESP), São Paulo, SP, Brazil (grant 2013/19473-6)
N/R
Grants #2014/00119-0 and 2015/14119-5 from the Research Funding Agency of São Paulo State (FAPESP), Brazil

Grants #2014/00119-0 and 2015/14119-5 from the Research Funding Agency of São Paulo State (FAPESP), Brazil
N/R
Research Foundation of the State of São Paulo (Grants # 2016/26154-2; 2015/08701-3) and CNPq, National Council for Scientific and Technological Development (Grant # 301102/2016-3).
N/R
No financial support

Research Funding Agency from São Paulo State (FAPESP), São Paulo, SP, Brazil (process #2013/13098-9)
No financial support
University of Kukurova research fund. Project: TSA-2018-10749

No financial support
No financial support
Department of Periodontology, School of Dentistry, Universidad Científica del Sur, Lima, Peru

FAI initiation (no. ID-0001465)
N/R

Grant from Kocaeli University Research Projects Grant 2016/028
São Paulo State (FAPESP), Brazil (grants #2018/03284-3; 2016/26154-2), Coordination for the Improvement of Higher Education Personnel (CAPES), Brazil—Finance Code 001, and National Council for Scientific and Technological Development from Brazil CNPq (grant # 304269/2019-0).

Geistlich Pharma, Wolhusen, Switzerland.
Research Funding Agency from São Paulo State (FAPESP), Brazil (grants #2018/03284-3); Coordination for the Improvement of Higher Education Personnel (CAPES), Brazil – Finance Code 001. National Council for Scientific and Technological Development from Brazil, CNPq (grant # 304269/2019-0)

No financial support
Deanship of Scientific Research at the Arab American University, Palestine
No financial support
N/R

No financial support

ticipants

Initial number of participants and distribution by groups	Drop-outs
22 patients<br>Control group n=22 GRD<br>Test group n=22 GRD	n=0
16 patients<br>Control group n=16 GRD<br>Test group n=16 GRD	n=0

15 patients<br>Control group n=15 GRD<br>Test group n=15 GRD	n=0
20 patients<br>Control group n=20 GRD<br>Test group n=20 GRD	n=3

17 patients<br>Control group n=17 GRD<br>Test group n=17 GRD	n=0
17 patients<br>Control group n=17 GRD<br>Test group n=17 GRD	n=0

15 patients<br>Control group n=15 GRD<br>Test group n=15 GRD	n=0
25 patients<br>Control group n=25 GRD<br>Test group n=25 GRD	n=0
85 patients<br>Control group n=43 GRD<br>Test group n=42 GRD	n=1 control group<br>n=1 test group

25 patients<br>(subsample from Cortellini et al. 2009)<br>Control group n=13 GRD<br>Test group n=12 GRD	n=0
30 patients<br>Control group n=30 GRD<br>Test group n=30 GRD	n=0
30 patients<br>Control group n=15 GRD<br>Test group n=15 GRD	n=0

10 patients<br>Control group n=10 GRD<br>Test group n=10 GRD	n=0
25 patients<br>Control group n=25 GRD<br>Test group n=25 GRD	n=0
25 patients<br>Control group n=25 GRD<br>Test group n=25 GRD	n=8
24 patients<br>Control group n=24 GRD<br>Test group n=24 GRD	n=0
20 patients<br>Control group n=10 GRD<br>Test group n=10 GRD	n=0

50 patients<br>Control group n=25 GRD<br>Test group n=25 GRD	n=0
29 patients<br>Control group n=14 GRD<br>Test group n=15 GRD	n=1 test group

29 patients<br>Control group n=14 GRD<br>Test group n=15 GRD	n=3 control group<br>n=2 test group
29 patients<br>Control group n=14 GRD<br>Test group n=15 GRD	n=4 control group<br>n=4 test group
80 patients<br>Control group n=42 GRD<br>Test group n=38 GRD	n=0
32 patients<br>Control group n=16 GRD<br>Test group n=16 GRD	n=1 control group

60 patients<br>Control group n=30 GRD<br>Test group n=30 GRD	n=0
50 patients<br>Control group n=25 GRD<br>Test group n=25 GRD	n=0
40 patients<br>Control group n=20 GRD<br>Test group n=20 GRD	n=0

40 patients<br>Control group n=20 GRD<br>Test group n=20 GRD	n=1 control group<br>n=3 test group
52 patients<br>Control group n=26 GRD<br>Test group n=26 GRD	n=0
36 patients<br>Control group n=18 GRD<br>Test group n=18 GRD	n=0

45 patients<br>Control group n=45 GRD<br>Test group n= 45 GRD	n=4
50 patients<br>Control group n=25 GRD<br>Test group n=25 GRD	n=0

68 patients<br>Control group n=17 GRD<br>Test group 1 n=17 GRD<br>Test group 2 n=17 GRD<br>Test group 3 n=17 GRD	n=0
50 patients<br>Control group n=25 GRD<br>Test group n=25 GRD	n=0
42 patients<br>Control group n=21 GRD<br>Test group n=21 GRD	n=0

42 patients<br>Control group n=21 GRD<br>Test group n=21 GRD	n=2 control group<br>n=1 test group
44 patients<br>Control group n=22 GRD<br>Test group n=22 GRD	n=2 control group<br>n=2 test group
40 patients<br>Control group n=20 GRD<br>Test group n=20 GRD	n=0
22 patients<br>Control group n=11 GRD<br>Test group n=11 GRD	n=0
19 patients<br>Control group n=19 GRD<br>Test group n=19 GRD	n=0

30 patients<br>Control group n=15 GRD<br>Test group n=15 GRD	n=0
30 patients<br>Control group n=15 GRD<br>Test group n=15 GRD	n=0
72 patients<br>Control group n=36 GRD<br>Test group n=36 GRD	n=5 control group<br>n=2 test group

22 patients<br>Control group n=22 GRD<br>Test group n=22 GRD	n=1 control group<br>n=1 test group
30 patients<br>Control group n=16 GRD<br>Test group n=14 GRD	n=0
48 patients<br>Control group n=24 GRD<br>Test group n=24 GRD	n=2 control group<br>n=4 test group

13 patients (contributing with 1, 2 or 3 recessions)<br>Control group n=17 GRD<br>Test group n=17 GRD	n=0
44 patients<br>Control group n=22 GRD<br>Test group n=22 GRD	n=2 control group

16 patients<br>Control group n=16 GRD<br>Test group n=16 GRD	n=0
62 patients<br>Control group n=31 GRD<br>Test group n=31 GRD	n=0

30 patients<br>Control group n=30 GRD<br>Test group n=30 GRD	n=0
75 patients<br>Control group n=25 GRD<br>Test groups n=50 GRD	n=0

38 patients<br>Control group n=19 GRD<br>Test group n=19 GRD	n=3 control group<br>n=3 test group
15 patients<br>Control group n=15 GRD<br>Test group n=15 GRD	n=0
60 patients<br>Control group n=30 GRD<br>Test group n=30 GRD	n=8<br>(n=4 drop-out in each group)
60 patients<br>Control group n=20 GRD<br>Test group 1 n=20 GRD<br>Test group 2 n=20 GRD	n=0

12 patients<br>Control group n=12 GRD<br>Test group n=12 GRD	n=0

**Follow-up** (Months)
6
6

12
12

6
30

6
6
6

108
6
6

6
12
60
12
12

12
6

36
120
12
6

12
12
6

24
6
12

12
12

6
6
6

24
12
12
12
12

12
18
6

12
12
48

6
12

6
12

6
6

12
6
6
6

6

Procedures
**Root coverage procedure(s)**
C: CAF + SCTG<br>T: CAF + ADM
C: CAF + SCTG<br>T: CAF + GTR

C: CAF + SCTG thick-tall<br>T: CAF + SCTG thin-short
C: CAF + SCTG<br>T: CAF + EMD

C: Envelope + SCTG (Advanced flap)
T: SCPF

C: Envelope + SCTG
T: SCPF

C: MCAF with vertical releasing incision + ADM<br>T: MCAF without vertical releasing incision + ADM
C: FGG<br>T: BCT
C: CAF<br>T: CAF + SCTG

C: CAF<br>T: CAF + SCTG
C: CAF + SCTG<br>T: CAF + b-TCP/rhPDGF-BB
C: CAF conventional + EMD<br>T: CAF microsurgical + EMD

C: CAF + SCTG<br>T: CAF + ADM
C: CAF + SCTG<br>T: CAF + CM
C: CAF + SCTG<br>T: CAF + CM
C: Envelope advanced + SCTG + no microscope<br>T: Envelope advanced + SCTG + microscope
C: CAF + SCTG<br>T: CAF + PPF

C: CAF + SCTG
T: LMCAF

C: CAF
T: CAF + SCTG

C: CAF<br>T: CAF + SCTG
C: CAF<br>T: CAF + SCTG
C: CAF + FDADM<br>T: CAF + SDADM
C: LPF<br>T: LALPF

C: CAF + SCTG (thickness ≥2mm, height equal to bone dehiscence)<br>T: CAF + SCTG (thickness <2mm, height 4mm)
C: CTG + SCTG<br>T: CAF + SCTG and LST removal
C: CAF + SCTG<br>T: CAF + SCTG + LLLT

C: CAF + SCTG<br>T: CAF + SCTG + LLLT
C: CTG + SCTG de-epithelialized with a blade<br>CTG + SCTG de-epithelialized via laser
C: CAF + SCTG - non-restored NCCL<br>T: CAF + SCTG + restored NCCL

C: CAF<br>T: CAF + CMX
C: CAF with a trapezoidal papillae<br>T: CAF with a triangular papillae

C: CAF<br>T1: CAF + CM<br>T2: CAF + EMD<br>T3: CAF + CM + EMD
C: LPF - No magnifying loupes, no microsurgical instruments and sutures<br>T: LPF - Magnifying loupes, microsurgical instruments and sutures
C: CAF + SCTG<br>T: TUN + SCTG

C: CAF + SCTG<br>T: TUN + SCTG
C: CAF via a split approach<br>T: CAF via a split-full-split
C: CAF + SCTG + NCCL odontoplasty<br>T: CAF + SCTG + NCCL restoration
C: LPF<br>T: LPF + ADM
C: CAF + SCTG<br>C: CAF + SCTG + EMD

C: SCPF
T: SCPF + EMD

C: CAF
T: CAF + HA

C: CAF + SCTG
T: CAF + SCTG + iPRF

C: CAF + SCTG
T: CAF + PCM

C: CAF
T: CAF + SCTG

C: CAF
T: CAF + SCTG

C: CAF<br>T: CAF + L-PRF
C: Envelope + SCTG<br>T: SCPF

C: CAF + SCTG<br>T: GUG (free gingival graft comprehending the marginal gingiva and the interdental papilla)
C: CAF + SCTG<br>T: CAF + CM

C: CAF + SCTG
T: CAF + VCMX

C: CAF
T1: CAF + CM
T2: CAF + XDM

C: CAF + SCTG positioned the gingival margin at the CEJ<br>T: CAF + SCTG positioned the gingival margin apical to the CEJ
C: CAF + SCTG<br>T: CAF + A-PRF
C: CAF + SCTG<br>T: CAF + PPF
C: CAF<br>T1: CAF + SCTG<br>T2: CAF + L-PRF

C: TUN + SCTG (Thin 1mm)
T: TUN + SCTG (Thick - 2mm)

Age distribution (Years)	Gender Distribution (Females/Males)	Inclusion of smokers
47.2±10.8 (24-67)	15/7	No
40.6±9.15 (30-54)	10/6	Use of any tobacco products within the last 30 days

(18-35)	N/R	N/R
44.9±11.6	10/10	No

33.5<br>(21-52)	11/6	No
33.5<br>(21-52)	11/6	No

23 to 54	6/9	No
50.6<br>(31.1-69.7)	17/8	No
C: 37.8±8.4<br>T: 35.0±8.7	C: 20/23<br>T: 28/14	Max 20 cigarettes<br>C: n=9 smokers<br>T: n=13 smokers

C: 51.1±7.5<br>T: 47.4±7.4	C: 5/8<br>T: 7/5	Max 20 cigarettes<br>C: n=2 smokers<br>T: n=1 smokers
43.8±10.7<br>(18-70)	26/4	NO current smokers<br>up to 6 months
(22- 44)	16/14	No

36.5±10.5<br>(21-53)	3/7	N/R
43.7±12.2<br>(18-73)	17/8	NO smokers for 6 months<br><br>n=12 never smoked and n=13 former smokers.
51.3±13.9	12/5	N/R
34.0±9.25<br>(18-55)	11/13	No
25.2±6.0<br>(16-40)	10/10	No

33.6±5.8<br>(20-45)<br><br>C: 32.2±5.5<br>(22–42)<br>T: 34.2±5.8<br>(21–41)	30/20	<10 cigarettes/day
C: 53.1±8.4<br>T: 45.9±10.3	C: 9/5<br>T: 13/2	Smoking ≤20 cigarettes/day

C: 53.1±8.4<br>T: 45.9±10.3	C: 9/5<br>T: 13/2	Smoking ≤20 cigarettes/day
C: 53.1±8.4<br>T: 45.9±10.3	C: 9/5<br>T: 13/2	Smoking ≤20 cigarettes/day
45.3<br>(23-74)<br><br>C: 47.4±14.0<br>T: 43.0±13.0	54/26<br><br>C: 26/16<br>T: 28/10	No
C: 29.3±4.8<br>T: 29.0±4.1	18/13<br>C: 8/7<br>T: 10/6	No

N/R	N/R	<10 cigarettes/day
N/R	N/R	<10 cigarettes/day
C: 41.36±8.81<br>T: 39.75±10.80	20/20<br><br>C: 10/10<br>T: 10/10	No

C: 42.17±8.90<br>T: 40.10±10.9	20/16<br>C: 10/7<br>T: 10/9	No
C: 27.5±5.3 (21 to 38)<br>T: 27.3±4.1 (22-36)	26/26<br><br>C: 14/12<br>T: 12/14	No
C: 38.9±11.3<br>T: 35.2±13.7	17/19<br><br>C: 8/10<br>T: 9/9	No

39.5±13.8 (20-73)	28/17	<10 cigarettes/day
N/R	N/R	<10 cigarettes/day

C: 38.12±12.95<br>T1: 39.47±10.94<br>T2: 39.29±10.23<br>T3: 33.24±10.41	42/26<br><br>C: 11/6<br>T1: 10/7<br>T2: 10/7<br>T3: 11/6	No
C: 29.4±4.2<br>T: 29.5±4.4	25/25<br><br>C: 11/8<br>T: 17/14	No
40.2±9.6<br>(24-59)<br><br>C: 40.7±8.9<br>T: 38.7±10.4	27/15<br><br>C: 12/9<br>T: 15/6	No

C: 40.9±9.0<br>T: 40.2±10.4	28/11<br><br>C: 14/5<br>T: 14/6	No
C: 36.4±12.0<br>T: 38.4±9.0	29/15<br>T: 15/7<br>C: 14/8	≤10 cigarettes/day
44.5±10.6<br>(22-60)<br><br>C: 45.3±11.3<br>T: 44.3±10.4	18/22<br><br>C: 10/10<br>T: 8/12	No
29.04±5.49<br>(21-39)	11/11	No
32.18±8.91<br>(18-55)	10/9	No

C: 28.8±9.96<br>T: 30.67±6.56	23/7<br><br>C: 11/4<br>T: 12/3	No
C: Median = 30.0±12.0<br>T: Median = 30.0±15.0	C: 7/8<br>T: 7/8	No
C: 37.5±12.6<br>T: 38.8±11.2	35/37	No

27.25±4.90<br>(20-45)	11/11	<20 cigarettes/day
C: 40.5±10.3<br>T: 37.7±9.4	22/8<br><br>C: 12/4<br>T: 10/4	≤10 cigarettes/day<br><br>C: 12% (n=2)<br>T: 29% (n=4)
C: 46.86±9.52<br>T: 44.6±11.93<br>(10-60)	20/22<br>C: 10/12<br>T: 10/10	No

41.7±9.11<br><br>C: 41.4±8.7<br>T: 41.5±9.6	9/4	No
36.21±10.15<br><br>C: 35.00 ±10.45<br>T: 37.32 ±9.99	37/5<br><br>C: 16/4<br>T: 21/1	No

Females: 41.7±10.1<br>Males: 34.8±8.8	10/6	N/R
47.6±10.0<br>(21-62)<br><br>C: 47.7±9.5<br>T: 46.1±9.9	29/33<br><br>C: 10/21<br>T: 19/12	No

50.7±11.4	19/11	No
44.2 ± 0.7<br>(19-66)<br><br>C: 45.0±12.5<br>T1: 43.6±12.1<br>T2: 44.1±10.4	51/24<br><br>C: 17/8<br>T1: 17/8<br>T2: 17/8	No

C: 32<br>(21-52)<br>T: 27<br>(19-50)	N/R	No
C: 34.68±8.6<br>T: 39.47±6.69	3/12	≤10 cigarettes
C: 40.08<br>(18-45)<br>T: 40.31<br>(18-45)	14/38	N/R
32.4±5<br>(18-47)	27/33	No

N/R	N/R	No

Stu

N. of cigarettes	History of periodontitis	Reason(s) for Treatment
0	N/R	N/R
N/R	N/R	N/R

N/R	No History of Periodontitis	Aesthetic
0	N/R	N/R

0	No History of Periodontitis	N/R
0	No History of Periodontitis	N/R

0	N/R	N/R
0	N/R	N/R
C: 0.9±2.8 pack/year<br>T: 1.8±4.3 pack/year	No active periodontitis	C: n=20 aesthetic, n=4 dental hypersensitivity, n=13 both, n=6 other reasons<br>T: n=15 aesthetic, n=4 dental hypersensitivity, n=14 both, n=9 other reasons

C: 1.3±2.5 pack/year<br>T: 0.7±1.5 pack/year	No active periodontitis	C: n=3 aesthetic, n=4 dental hypersensitivity, n=3 both, n=4 other reasons<br>T: n=2 aesthetic, n=1 dental hypersensitivity, n=2 both, n=7 other reasons
n=14 patients had previously smoked 11.0±11.4 cigarettes for 6.6±6.6 years	N/R	N/R
0	N/R	Root sensitivity<br>Aesthetic problem

N/R	N/R	N/R
N/R	N/R	N/R
N/R	N/R	N/R
No	No	N/R
0	N/R	N/R

N/R	N/R	N/R
C: n=7 smokers (5.2 ± 5.9 cigarettes/day)<br>T: n=5 smokers (4.7 ± 6.9 cigarettes/day)	NO active periodontitis , no site showing PD>4mm	Aethetic<br>Sensitivity

C: n=7 smokers (5.2 ± 5.9 cigarettes/day)<br>T: n=5 smokers (4.7 ± 6.9 cigarettes/day)	NO active periodontitis , no site showing PD>4mm	Aethetic Sensitivity
C: n=7 smokers (5.2 ± 5.9 cigarettes/day)<br>T: n=5 smokers (4.7 ± 6.9 cigarettes/day)	NO active periodontitis , no site showing PD>4mm	Aethetic Sensitivity
0	N/R	N/R
0	N/R	N/R

N/R	N/R	N/R
N/R	N/R	N/R
0	No active periodontitis	N/R

0	No active periodontitis	N/R
0	No History of periodontitis	N/R
0	N/R	N/R

N/R	N/R	N/R
N/R	N/R	Aesthetic

0	No active periodontitis	Aesthetic<br>Root sensitivity
0	No History of periodontitis	N/R
0	N/R	Aesthetic<br>Root sensitivity

0	N/R	Aesthetic<br>Root sensitivity
N/R	N/R	N/R
0	N/R	Aesthetic<br>Root sensitivity
0	N/R	N/R
0	N/R	Aesthetic<br>Root sensitivity

0	N/R	N/R
0	No active periodontitis	Aesthetic<br>Root sensitivity
0	No active periodontitis	N/R

N/R	N/R	N/R
	No active periodontitis	Aesthetic<br>Root sensitivity
0	No active periodontitis	Aesthetic<br>Root sensitivity

0	No active periodontitis	N/R
0	N/R	N/R

N/R	No active periodontitis	N/R
0	No active periodontitis	Aesthetic<br>Root sensitivity

0	No active periodontitis	N/R
0	No active periodontitis	Aesthetic<br>Root sensitivity

0	N/R	N/R
N/R	N/R	N/R
N/R	No active periodontitis	N/R
0	N/R	Patients concern for progression, aesthetic or hypersensitivity

0	No History of periodontitis	N/R

udy Subjects

Recession and tooth type	REC depth
Miller I and II<br><br>N/R	C: 3.0±0.7<br>T: 2.5±0.8
Miller I and II<br><br>N/R	C: 3.4±1.0<br>T: 3.7±1.1

Miller I and II<br><br>Maxillary non-molar teeth	C: 3.9±0.80<br>T: 4.0±0.76
Miller II<br><br>n=1 mandibular and n=19 maxillary incisors or premolar teeth	C: 4.25±0.72<br>T: 4.25±0.44

Miller I<br><br>Maxillary canines and premolars	C: 2.15±0.59<br>T: 2.20±0.56
Miller I<br><br>Maxillary canines and premolars	C: 2.15±0.59<br>T: 2.20±0.56

Miller I and II<br><br>n=10 maxillary canines, n=10 maxillary premolars, n=2 mandibular canines, and n=8 mandibular premolars	C: 2.73±0.76<br>T: 2.88±0.81
N/R	C: 2.47±0.46<br>T: 2.44±0.46
Miller I and II<br><br>C: n=3 maxillary incisor, n=26 maxillary canines and n=14 maxillary premolars<br>T: n=1 maxillary incisor, n=20 maxillary canines and n=21 maxillary premolars	C: 2.4±0.7<br>T: 2.7±0.7

Miller I and II<br><br>C: n=1 maxillay incisor, 1n=0 maxillary canines and 2n= maxillary premolars<br>T: n=3 maxillary canines and n=9 maxillary premolars	C: 2.4±1.0<br>T: 2.4±0.8
Miller class II<br><br>Non-molar teeth	C: 3.4±0.41<br>T: 3.2±0.41
Miller I and III<br><br>C: n=2 maxillary and n=6 mandibular canines, n=4 maxillary and n=1 mandibular premolars, n=1 maxillary lateral incisors<br>T: n=5 maxillary and n=1 mandibular canine, n=2 maxillary and n=3 mandibular premolars, n=2 maxillary lateral incisors and n=2 maxillary central incisors	C: 2.47±0.49<br>T: 2.40±0.47

Miller I and II<br><br>Maxillary canines and premolars	C: 2.8±0.79<br>T: 2.3±0.48
N/R<br><br>Maxillary and mandibular non-molar teeth	C: 3.2±0.35<br>T: 3.14±0.23
Miller I and II<br><br>Maxillary and mandibular non-molar teeth	N/R
Miller I and II<br><br>n=26 maxillary canines<br>n=22 maxillary premolars	C: 2.53±0.55<br>T: 2.51±0.35
Miller I and II<br><br>Anterior maxillary and mandibular teeth	C: 3.3±0.95<br>T: 3.6±0.84

Miller I and II<br><br>C: n=6 mandible and n=19 maxillary molars<br>T: n=7 mandible and n=18 maxillary molars	C: 5.0±1.08<br>T: 4.56±1.04
RT2<br><br>C: n=3 maxillary incisors, n=11 maxillary canines<br>T: n=2 maxillary incisors, n=8 maxillary canines, n=5 maxillary premolars	C: 2.6±0.6<br>T: 2.9±0.7

RT2<br><br>Maxillary teeth	C: 2.6±0.6<br>T: 2.9±0.7
RT2<br><br>Maxillary teeth	C: 2.6±0.6<br>T: 2.9±0.7
Miller I and II<br>C: n=37 Miller I, n=5 Miller II<br>T: n=32 Miller I, n=6 Miller II<br><br>C: n=16 maxillary premolars, n=22 maxillary canines, n=4 maxillary incisors<br>T: n=16 maxillary premolars, n=19 maxillary canines, n=3 maxillary incisors	C: 2.73±0.71<br>T: 2.91±1.01
Miller II<br><br>C: n=4 mandibular central incisors, n=7 mandibular lateral incisors, n=4 mandibular canines<br>T: n=6 mandibular central incisors, n=7 mandibular lateral incisors, n=3 mandibular canines	C: 4.7±0.9<br>T: 5.0±0.6

Miller I and II<br><br>Maxillary teeth	C: 3.93±0.67<br>T: 3.80±0.96
Miller I and II<br><br>Mandibular teeth	C: 3.72±0.84<br>T: 3.76±1.01
Miller I and II<br><br>C: n=15 maxillary canines, n=5 maxillary premolars<br>T: n=12 maxillary canines, n=8 maxillary premolars	C: 3.33±0.72<br>T: 3.09±0.67

Miller I and II<br><br>C: n=12 maxillary canines, n=5 maxillary premolars<br>T: n=12 maxillary canines, n=7 maxillary premolars	C: 3.33±0.72<br>T: 3.09±0.67
RT1<br><br>C: n=18 maxillary incisors, n=8 maxillary canines<br>T: n=20 maxillary incisors, n=6 maxillary canines	C: 4.4±1.2<br>T: 4.6±1.1
Miller I and II<br><br>C: n=6 maxillary premolars, n=12 maxillary canines<br>T: n=5 maxillary premolars, n=13 maxillary canines	C: 3.39±0.57<br>T: 3.73±0.5

Miller I and II<br><br>n=2 maxillary central incisors, n=40 maxillary canines, n=24 maxillary premolars and n=4 maxillary first molars<br>n=2 mandibular incisors, n=4 mandibular canines and mandibular n=14 pre-molars	C: 3.34±1.00<br>T: 3.46±0.90
Miller I and II<br><br>Maxillary teeth excluding molars	C: 3.33±0.80<br>T: 3.23±0.86

Miller I and II<br><br>C: n=6 maxillary canines, n=11 maxillary premolars<br>T1: n=9 maxillary canines, n=8 maxillary premolars<br>T2: n=7 maxillary canines, n=10 maxillary premolars<br>T3: n=11 maxillary canines, n=6 maxillary premolars	C: 3.22±0.45<br>T1: 3.12±0.40<br>T2: 3.04±0.31<br>T3: 3.16±0.33
Miller III<br><br>Anterior mandibular and maxillary teeth	C: 4.24±0.88<br>T: 4.4±1.08
Miller I and II<br><br>C: n=14 maxillary canines, n=7 maxillary premolars<br>T: n=11 maxillary canines, n=10 maxillary premolars	C: 3.2±0.7<br>T: 3.0±0.6

C: n=14 maxillary canines, n=5 maxillary premolars<br>T: n=11 maxillary canines, n=9 maxillary premolars	C: 3.2±0.7<br>T: 3.0±0.6
Miller I<br><br>C: n=3 maxillary incisors, n=10 maxillary canines, n=9 maxillary premolars<br>T: n=2 maxillary incisors, n=12 maxillary canines, n=8 maxillary premolars	C: 2.33±0.9<br>T: 2.47±0.9
Miller I and II<br><br>C: n=11 maxillary canines, n=9 maxillary premolars<br>T: n=7 maxillary canines, n=13 maxillary premolars	C: 3.3±0.7<br>T: 3.3±1.0
Miller I and II<br><br>Non-molar teeth	C: 4.04±1.13<br>T: 4.0±0.63
Miller I and II<br><br>Maxillary canines	C: 4.04±1.42<br>T: 3.91±1.20

N/R	C: 2.32±0.21<br>T: 2.18±0.13
RT1/Miller I<br><br>C: n=5 maxillary canines, n=7 maxillary premolars, n=1 mandibular canines and n=2 mandibular premolars<br>T: n=2 maxillary canines, n=7 maxillary premolars, n=2 mandibular canines and n=4 mandibular premolars	Median<br>C: 3.0±1.0<br>T: 3.0±1.0
Miller I and II<br><br>Incisors and canines	C: 4.0±1.0<br>T: 3.9±0.8

Miller I and II	C: 2.55±0.69<br>T: 2.67±0.65
RT1<br><br>C: n=1 maxillary incisor, n=7 maxillary canines, n=8 maxillary premolars<br>C: n=6 maxillary canines, n=7 maxillary premolars, n=1 maxillary molar	C: 3.2±0.5<br>T: 3.4±0.6
RT1<br><br>C: n=4 maxillary canines, n=18 maxillary premolars<br>T: n=2 maxillary incisors, n=5 maxillary canines, n=13 maxillary premolars	C: 3.45±1.01<br>T: 3.2±0.77

Miller I and II<br><br>Non-molar teeth<br>C: n=5 incisors, n=7 canines, n=5 premolars<br>T: n=2 incisors, n=8 canines, n=7 premolars	Analysis as a parallel group:<br>C: 3.2±0.7<br>T: 3.3±0.5<br><br>Analysis as a split-mouth group:<br>C: 3.2±0.8<br>T: 3.3±0.5
Miller Class I<br><br>C: n=4 maxillary incisors, n=2 maxillary canines, n=14 maxillary premolars<br>T: n=3 maxillary incisors, n=6 maxillary canines, n=13 maxillary premolars	C: 1.54±0.91<br>T: 1.58±0.96

RT1<br><br>Non-molar teeth<br>C: n=1 incisor, n=10 canines, n=5 premolars<br>T: n=1 incisor, n=7 canines, n=8 premolars	C: 3.00±0.57<br>T: 3.06±0.54
RT1<br>C: n=12 Canines, n=19 Premolars<br>T: n=12 Canines, n=19 Premolars	C: 3.5±0.8<br>T: 3.7±0.7

Miller I and II<br><br>Non-molar teeth	C: 3.73±0.95<br>T: 3.63±0.79
RT1<br><br>n=27 canines, and n=38 premolars	C: 3.1±0.5<br>T1: 3.2±0.7<br>T2: 3.3±0.9

RT1<br><br>Maxillary and mandibular incisors and canines	C: 3.6±1.5<br>T: 4.2±2.2
Miller's class I and II<br>maxillary canines or premolars	C: 2.87±0.83<br>T: 2.53±0.74
Miller I and II N/R	C: 3.15±1.62<br>T: 2.38±0.65
RT1<br><br>C: n=5 maxillary incisors, n=8 maxillary canines, n=7 maxillary premolars<br>T1: n=6 maxillary incisors, n=7 maxillary canines, n=7 maxillary premolars<br>T2: n=6 maxillary incisors, n=8 maxillary canines, n=6 maxillary premolars	C: 4.00±0.65<br>T1: 4.35±0.43<br>T2: 4.08±0.47

RT1<br><br>Maxillary teeth	C: 1.93±0.64<br>T: 2.02±0.89

REC width	KT width	Gingival thickness (GT)
C: 3.5±0.7<br>T: 3.8±0.7	C: 1.8±1.2<br>T: 1.7±0.7	N/R
C: 3.5±0.8<br>T: 3.4±0.9	C: 2.5±1.2<br>T: 2.8±1.4	N/R

N/R	C: 1.2±0.41<br>T: 1.1±0.26	N/R
C: 6.40±1.57<br>T: 6.10±1.68	C: 2.50±0.89<br>T: 2.40±0.77	N/R

C: 3.58±0.67<br>T: 3.74±0.68	C: 3.30±0.81<br>T: 3.52±0.62	C: 1.01±0.17<br>T: 1.04±0.26<br>measured 2mm apical to the GM
C: 3.58±0.67<br>T: 3.74±0.68	C: 3.30±0.81<br>T: 3.52±0.62	C: 1.01±0.17<br>T: 1.04±0.26<br>measured 2mm apical to the GM

N/R	C: 2.17±1.42<br>T: 1.94±1.66	C: 0.55±0.21<br>T: 0.48±0.29
N/R	C: 1.17± .58<br>T: 1.07±0.58	N/R
C: 3.8±0.7<br>T: 3.8±0.8	C: 3.2±1.3<br>T: 2.7±1.2	N/R

N/R	C: 3.8±1.5<br>T: 3.2±1.0	N/R
C: 4.0±0.41<br>T: 4.0±0.41	C: 1.9±0.8<br>T: 1.9±0.6	N/R
N/R	C: 2.02±1.17<br>T: 2.23±0.69	C: 1.01±0.51<br>T: 0.80±0.39<br>measured 1mm coronally to the MGJ

N/R	C: 2.5±0.57<br>T:3.1±0.89	N/R
C: 4.3±0.6<br>T: 4.06±0.49	C: 2.78±1.35<br>T: 2.44±1.02	N/R
N/R	N/R	N/R
C: 3.87±0.79<br>T: 3.80±0.79	C: 2.66±1.20<br>T: 2.45±0.99	C: 0.97±0.18<br>T: 0.89±0.23<br>measured 2mm apical to the GM
N/R	C: 3.1±0.57<br>T: 3.5±0.53	N/R

N/R	C: 0.52±0.5<br>T: 0.68±0.62	C: 0.77±0.32<br>T: 0.84±0.22<br>measured 1.5mm apical to the gingival margin
N/R	C: 2.8±0.8<br>T: 2.6±1.0	N/R

N/R	C: 2.8±0.8<br>T: 2.6±1.0	N/R
N/R	C: 2.8±0.8<br>T: 2.6±1.0	N/R
C: 3.49±0.99<br>T: 3.47±0.73	C: 3.29±1.49<br>T: 2.93±1.76	C: 1.21±0.56<br>T: 1.30±0.47<br>measured 1mm apical to the GM<br><br>C: 0.74±0.86<br>T: 1.12±0.87<br>measured 3mm apical to the GM
C: 3.1±0.7<br>T: 3.1±0.8	C: 1.5±0.5<br>T: 1.5±0.5	N/R

N/R	C: 1.13±0.73<br>T: 1.33±0.71	C: 0.72±0.13<br>T: 0.75±0.15<br>measured 1.5mm apical to the GM
N/R	C: 0.64±0.57<br>T: 0.52±0.51	C: 0.43±0.11<br>T: 0.42±0.11
N/R	C: 3.31±1.00<br>T: 3.75±1.01	C: 1.26±0.30<br>T: 1.48±0.40<br>measured at midpoint between GM and MGJ

N/R	C: 3.31±1.00<br>T: 3.75±1.01	C: 1.26±0.30<br>T: 1.48±0.40<br>measured at midpoint between GM and MGJ
C: 3.2±1.0<br>T: 3.1±0.8	C: 1.5±0.5<br>T: 1.3±0.5	N/R
N/R	C: 2.41±1.2<br>T: 2.27±0.86	C: 0.9±0.23<br>T: 0.9±0.24<br>measured at mid-point from GM to MGJ

C: 4.10±0.93<br>T: 4.08±0.89	C: 2.00±1.22<br>T: 1.97±1.13	C: 0.89±0.34<br>T: 0.89±0.28<br>measured 1 mm apical from the GM
N/R	C: 1.43±0.50<br>T: 1.36±0.49	N/R

N/R	C: 2.86±1.30<br>T1: 2.58±1.47<br>T2: 2.59±1.43<br>T3: 2.32±1.07	C: 0.94±0.30<br>T1: 0.84±0.26<br>T2: 0.88±0.26<br>T3: 0.92±0.19<br>measured at midpoint from GM to MGJ
C: 3.8±0.50<br>T: 3.96±0.46	C: 0.72±0.54<br>T: 0.64±0.57	N/R
N/R	C: 2.8±0.9<br>T: 2.6±1.1	C: 1.0±0.3<br>T: 1.0±0.4

N/R	C: 2.8±0.9<br>T: 2.6±1.1	C: 1.0±0.3<br>T: 1.0±0.4
N/R	C: 2.6±1.0<br>T: 2.5±0.7	C: 0.9±0.1<br>T: 0.9±0.2<br>measured 3mm apical to the GM
N/R	C: 2.9±0.9<br>T: 2.7±1.3	C: 0.9±0.2<br>T: 1.0±0.5
C: 3.41±0.54<br>T: 3.45±0.52	C: 0.73±0.41<br>T: 0.77±0.34	C: 0.72±0.09<br>T: 0.70±0.11
C: 3.73±0.75<br>T: 3.73±0.91	N/R	N/R

N/R	C: 3.23±0.88<br>T: 3.32±0.71	C: 1.11±0.16<br>T: 1.13±0.21<br>measured at midpoint between GM and MGJ
N/R	Median<br>C: 2.0±1.0<br>T 2.0±1.0	N/R
C: 3.8±1.1<br>T: 4.2±1.3	C: 2.0±1.1<br>T: 2.0±1.3	C: 0.9±0.3<br>T: 0.8±0.3<br>1mm apical to the GM

N/R	C: 2.20±0.61<br>T: 2.17±0.65	N/R
N/R	C: 3.1±0.5<br>T: 2.9±1.1	C: 0.80±0.09<br>T: 0.78±0.12
N/R	N/R	N/R

Analysis as a parallel group:<br>C: 3.6±1.0<br>T: 4.2±1.2<br><br>Analysis as a split-mouth group:<br>C: 3.6±1.0<br>T: 4.2±1.3	Analysis as a parallel group:<br>C: 4.1±1.3<br>T: 3.3±1.0<br><br>Analysis as a split-mouth group:<br>C: 4.2±1.4<br>T: 3.4±0.9	Analysis as a parallel group:<br>C: 1.5±0.5<br>T: 1.7±0.9<br><br>Analysis as a split-mouth group:<br>C: 1.6±0.5<br>T: 1.6±0.9
C: 2.89±1.43<br>T: 2.89±1.69	C: 3.35±1.93<br>T: 4.27±1.02	C: 1.08±0.37<br>T: 1.07±0.44

C: 3.81±0.65<br>T: 3.90±0.68	C: 2.03±1.10<br>T: 1.71±1.48	N/R
N/R	C: 2.1±1.0<br>T: 2.4±1.5	C: 1.0±0.5<br>T: 1.0±0.6

C: 3.7±0.48<br>T: 3.7±0.64	C: 2.3±0.88<br>T: 2.5±1.25	C: 158.37 ± 72.89<br>T: 189.40 ± 73.87<br>measure in mm3 using a digital impression<br><br>Tissue Phenotype<br>C: Thin: 15, Thick: 15<br>T: Thin: 17, Thick: 13
N/R	C: 2.3±1.2<br>T1: 2.8±1.4<br>T2: 2.4±1.5	C: 1.0±0.3<br>T2: 1.0±0.3<br>T2 1.0±0.3<br>measured 1.5mm apical from the GM

C: 3.9±1.1<br>T: 4.4±1.0	C: 2.1±1.2<br>T: 1.4±0.9	C: 0.9±0.3<br>T: 0.8±0.2
C: 3.87±0.51<br>T: 3.40±0.63	C: 3.47±0.64<br>T: 3.20±0.67	N/R
C: 3.11±0.76<br>T: 3.30±0.85	C: 2.57±0.57<br>T: 3.73±1.36	N/R
N/R	C: 1.65±0.59<br>T1: 1.85±0.67<br>T2: 1.95±0.51	C: 1.07±0.44<br>T1: 0.96±0.26<br>T2: 1.05±0.32

N/R	N/R	C: 0.88±0.19<br>T: 0.88±0.19

NCCL	
	Scoring system (e.g., RES)
N/R	N/R
N/R	N/R

N/R	N/R
N/R	N/R

NO	N/R
NO	N/R

N/R	Esthetic scoring taking into account the root coverage, the gingival anatomy, contour and colour of the tissue
N/R	N/R
Identifiable CEJ<br>Horizontal step <1mm	N/R

Identifiable CEJ<br>Horizontal step <1mm	N/R
N/R	N/R
NO	N/R

N/R	RES
N/R	N/R
N/R	N/R
N/R	N/R
N/R	N/R

NO	N/R
Teeth presenting with root steps >1 mm at CEJ level and/or presence of a root/crown abrasion or with crowns or restorations at the Cement- to-Enamel Junction (CEJ) were excluded	RES

Teeth presenting with root steps >1 mm at CEJ level and/or presence of a root/crown abrasion or with crowns or restorations at the Cement- to-Enamel Junction (CEJ) were excluded	RES
Teeth presenting with root steps >1 mm at CEJ level and/or presence of a root/crown abrasion or with crowns or restorations at the Cement- to-Enamel Junction (CEJ) were excluded	RES
N/R	N/R
Presence of a step <2 mm at CEJ level and/or the presence of a root abrasion, but with an identifiable CEJ	RES

Presence of identifiable CEJ (a step ≤1 mm at CEJ level and/or presence of a root abrasion, but with an identifiable CEJ, were accepted	N/R
Presence of identifiable CEJ (a step ≤1 mm at CEJ level and/or presence of a root abrasion, but with an identifiable CEJ, were accepted	N/R
Identifiable CEJ	RES, and QCE

Identifiable CEJ	RES, and QCE
Identifiable buccal CEJ; presence of a step ≤1 mm at the CEJ level and/or the presence of a root abrasion, but with an identifiable CEJ	N/R
B+	MRES

Visible CEJ	RES
Identifiable CEJ, a step ≤1 at CEJ and/or presence of a root abrasion were accepted	N/R

Identifiable CEJ	N/R
Identifiable CEJ	RES
Visible CEJ	RES

Visible CEJ	RES
Identifiable CEJ	N/R
B+	MRES
N/R	RES
NO	RES

N/R	QCE
Restored NCCL admitted	N/R
Identifiable CEJ	Aesthetic VAS

N/R	N/R
NCCL yes excluding severe horizontal step in the root area at experimental site (>2 mm)<br><br>C: B+ 88% (n=14); B- 12% (n=2)<br>T: B+ 93% (n=13); B- 7% (n=1)	RES
N/R	RES

NO	RES
Identifiable CEJ	N/R

N/R	N/R
Class B (B+ or B-), partially restored with resin composite	MRES<br>(Modified score for CRC from 0 to 3 and added the colour of the lesion/cervical restoration from 0=not match to 3=good colour).<br>The final score goes from 0 to 10

Visible CEJ	N/R
Identifiable or restored CEJ	RES

Identifiable CEJ	RES
N/R	RES
N/R	N/R
N/R	RES

NO	RES

Overall score	CRC (0=failure, 3=partial, 6=complete)
4 point scale<br>C: excellent: n=7, good: n=12, fair: n=3, poor: n=0<br><br>T: excellent: n=18, good: n=4, fair: n=0, poor: n=0	N/R
N/R	N/R

N/R	N/R
N/R	N/R

N/R	N/R
N/R	N/R

N/R	N/R
N/R	N/R
N/R	N/R

N/R	N/R
N/R	N/R
N/R	N/R

C: 7.9±2.3<br>T: 8.1±2.3	C: 4.8±1.5<br>T: 5.1±1.4
N/R	N/R
N/R	N/R
N/R	N/R
N/R	N/R

N/R	Root coverage VAS (0=bad, average=50 and excellent=100)<br>C: 94.0±6.5<br>T: 85.2±7.1
C: 6.7±1.5<br>T: 7.6±1.7	C: 3.86±1.4<br>T: 4.71±1.54

C: 7.5 1.8<br>T: 8.0±1.5	C: 4.1±1.55<br>T: 5.1±1.41
C: 7.0±1.7<br>T: 7.6±1.4	C: 3.6±1.3<br>T: 4.9±1.5
N/R	N/R
C: 7.1±1.0<br>T: 8.8±1.2	N/R

N/R	Root coverage VAS (0=very bad, 50=average, 100=excellent)<br>C: 79.7 ± 11.0<br>T: 83.7 ± 11.3
N/R	Root coverage<br>VAS 100<br>C: 8.96±1.62<br>T: 9.76±0.83
C: 7.85±0.95<br>T: 8.05±1.09	N/R

C: 8.68±0.97<br>T: 8.52±1.21	N/R
N/R	N/R
C: 7.44±2.3<br>T: 7.52±2.27	Modified 0 to 3<br><br>C: 1.83±1.5<br>T: 1.58±1.5

C: 7.34±2.90<br>T: 7.85±2.42	C: 4.54±1.79<br>T: 4.61±2.02
N/R	Root coverage VAS 100<br><br>6 months<br>C: 92.0±9.97<br>T: 91.0±12.4<br><br>12 months

N/R	N/R
C: 8.4±1.35<br>T: 9.24±0.93	N/R
C: 8.4±1.5<br>T: 7.8±1.9	C: 5.1±1.3<br>T: 4.1±1.7

C: 8.7±1.2<br>T: 8.4±1.6	C:5.5±1.1<br>T: 4.7±1.5
N/R	N/R
C: 6.65±2.5<br>T: 7.73±1.7	Modified 0 to 3<br><br>C: 1.05±1.4<br>T: 1.1±1.4
C: 7.36±2.11<br>T: 9.00±1.73	C: 4.36±1.56<br>T: 5.18±1.4
C: 8.37±1.85<br>T: 8.93±1.43	C: 4.68±1.53<br>T: 5.0±1.46

C: 2.85±0.79<br>T: 2.57±0.63	N/R
N/R	N/R
C: 9.2±1.3<br>T: 9.6±0.8	N/R

N/R	N/R
C: 7.7±1.8<br>T: 8.3±1.8	C: 4.4±1.7<br>T: 5.1±1.4
C: 9.14±1.08<br>T: 7.25±1.29	C: 5.73±0.88<br>T: 4.95±1.47

Analysis as a parallel group:<br>C: 8.3±1.5<br>T: 6.9±1.5<br><br>Analysis as a split-mouth group:<br>C: 8.2±1.6<br>T: 7.0±1.4	N/R
N/R	N/R

N/R	N/R
C: 8.1±1.8<br>T: 8.6±2.0	Modified 0 to 3<br><br>C: 2±1.4<br>T: 2.1±1.4

N/R	N/R
C: 6.9±1.6<br>T1: 6.9±2.1<br>T2: 6.6±1.6	C: 3.6±1.5<br>T1: 3.3±1.9<br>T2: 3.2±1.2

6 months:<br>C: 7.9±1.4<br>T: 9.3±1.3<br><br>12 months<br>C: 7.4±1.3<br>T: 9.2±1.4	N/R
C: 9.40±1.24<br>T: 8.27±1.43	N/R
N/R	N/R
C: 7.90±1.41<br>T1: 8.20±1.61<br>T2: 7.95±1.57	N/R

C: 8.25±1.86<br>T: 7.00±2.39	N/R

PROFESSIONALLY DETERMINED AESTHET	
Marginal tissue contour (0= irregular, 1=proper)	Soft tissue texture (0=scar, 1=absence of scar)
Excellent result for gingival contour<br>C: 72.7 (n=16)<br>T: 95.45 (n=21)	Excellent results for tissue consistency<br>C: 90.9 (n=20)<br>T: 95.45 (n=21)<br><br>Excellent results for contiguity with adjacent tissue<br>C: 27.3 (n=6)<br>T: 77.3 (n=17)
Contour<br>C: good n=13, poor n=1, irregular n=2<br>T: good n=15, poor n=0, irregular n=1	Consistency<br>C: firm n=16, spongy n=0<br>T: firm n=16, spongy n=0<br><br>Contiguity or blending<br>C: yes n=14, no n=2<br>T: yes n=16, no n=0

N/R	N/R
N/R	N/R

N/R	N/R
N/R	N/R

N/R	N/R
N/R	N/R
N/R	N/R

N/R	N/R
N/R	More firm Less firm, Equally firm<br>C: equally firm 100%<br>T: C: equally firm 100%
N/R	N/R

C: 0.8±0.4<br>T: 0.8±0.4	C: 0.5±0.5<br>T: 0.5±0.5
Six-Months Evaluation<br><br>Texture matches to surrounding tissue<br><br>C: less firm 0% (n=0), equally firm 52.9% (n=9), more firm 47.1% (n=8)<br>T: less firm 35.3% (n=6), equally firm 58.8% (n=10), more firm 5.9% (n=1)	N/R
Texture matches to surrounding tissue<br><br>C: less firm 0% (n=0), equally firm 35.3% (n=6), more firm 64.7% (n=11)<br>T: less firm 0% (n=0), equally firm 88.2% (n=15), more firm 11.8% (n=2)	N/R
N/R	N/R
N/R	N/R

N/R	N/R
C: 0.93±0.27<br>T: 0.93±0.27	C: 0.43±0.51<br>T: 0.29±0.47

C: 0.82± .42<br>T: 0.92±0.27	C: 1.0±0.0<br>T: 0.54±0.51
C: 0.8±0.42<br>T: 1.0±0.0	C: 0.9±0.32<br>T: 0.45±0.5
N/R	N/R
N/R	N/R

N/R	N/R
N/R	N/R
N/R	N/R

N/R	N/R
N/R	N/R
C: 0.44±0.5<br>T: 0.88±0.3	C: 0.77±0.4<br>T: 0.52±0.5

C: 0.71±0.46<br>T: 0.780.42	C: 0.61±0.49<br>T: 0.51±0.51
N/R	Contiguity present/absent (yes)<br><br>6 months<br>C: 68% (n=17)<br>T: 20% (n=5)

N/R	N/R
N/R	N/R
C: 0.8±0.4<br>T: 0.9±0.3	C: 0.4±0.5<br>T: 0.8±0.3

C: 0.8±0.4<br>T: 0.9±0.3	C: 0.7±0.4<br>T: 0.8±0.4
N/R	N/R
C: 0.6±0.5<br>T: 0.94±0.2	C: 0.75±0.4<br>T: 0.84±0.3
C: 0.36±0.5<br>T: 0.82±0.4	C: 0.73±0.47<br>T: 1.0±0.0
C: 0.98±1.25<br>T: 1.0	C: 0.81±0.4<br>T: 0.93±0.25

N/R	N/R
N/R	N/R
N/R	N/R

N/R	N/R
C: 0.94±0.25<br>T: 0.93±0.27	C: 0.56±0.51<br>T: 0.57±0.51
C: 1.0±0.0<br>T: 0.35 ±0.49	C: 0.77±0.43<br>T: 0.65±0.49

N/R	N/R
N/R	N/R

N/R	N/R
C: 0.8±0.3<br>T: 0.9±0.2	C: 0.8±0.3<br>T: 0.87±0.3

N/R	1= firmer, 2= less firm, 3= equally firm<br><br>Baseline:<br>C: firmer: n=0, less firm: n=3, equally firm: n=27<br>T: firmer: n=1, less firm: n=2, equally firm: n=27<br><br>12 months<br>C: firmer: n=2, less firm: n=12, equally firm: n=16<br>T: firmer: n=1, less firm: n=10, equally firm: n=19
C: 0.8±0.4<br>T1: 0.9±0.2<br>T2: 0.7±0.4	C: 0.6±0.5<br>T1: 0.7±0.4<br>T2: 0.6±0.4

N/R	N/R
N/R	N/R
N/R	N/R
N/R	N/R

N/R	N/R

TIC SCORES

Mucogingival junction (0=Not aligned, 1=Aligned)	Gingival color (0=different from adjacent tissues, 1=matching)
N/R	Excellent results for colour<br>C: 31.8 (n=7)<br>T: 81.8 (n=18)
N/R	Colour match<br>C: excellent n=11, good n=5, adequate n=0, Unsatisfactory n=0<br>T: excellent n=15, good n=0, adequate n=1, Unsatisfactory n=0

N/R	N/R
N/R	N/R

N/R	N/R
N/R	N/R

N/R	N/R
N/R	N/R
N/R	N/R

N/R	N/R
N/R	More red, Less red, Equally red<br>C: equally red 90% (n=27)<br>T: equally red 100% (n=30)
N/R	N/R

C: 0.9±0.3<br>T: 0.8±0.4	C: 0.9±0.3<br>T: 0.9±0.3
N/R	Six-Months evaluation<br><br>Colour match to surrounding tissue<br><br>C: Less red 0% (n=0), Equally red 70.6% (n=12), More red 29.4% (n=5)<br>T: Less red 0% (n=0), Equally red 64.7% (n=11), More red 35.3% (n=6)
N/R	Colour match to surrounding tissue<br><br>C: Less red 11.8% (n=2), Equally red 82.4% (n=14), More red 5.9% (n=1)<br>T: Less red 0% (n=0), Equally red 88.2% (n=15), More red 11.8% (n=2)
N/R	N/R
N/R	N/R

N/R	Colour match VAS (0=bad, average=50 and excellent=100)<br>C: 93.2±8.0<br>T: 95.6±5.8
C: 0.5±0.51<br>T: 0.78±0.43	C: 1.0±0.0<br>T: 0.86±0.36

C: 0.73±0.42<br>T: 0.54±0.52	C: 0.91± .32<br>T: 0.92±0.27
C: 0.7±0.48<br>T: 0.45±0.52	C: 1.0±0.0<br>T: 0.82±0.4
N/R	N/R
N/R	N/R

N/R	Colour match VAS (0=very bad, 50=average, 100=excellent)<br>C: 72.7±11.7<br>T: 79.0±10.3
N/R	Colour match VAS 100<br>C: 6.72±2.03<br>T: 8.16±1.86
N/R	N/R

N/R	N/R
N/R	N/R
C: 0.72±0.4<br>T: 0.70±0.5	C: 1±0<br>T: 1±0

C: 0.76±0.43<br>T: 0.80±0.40	C: 0.90±0.30<br>T: 0.90±0.16
N/R	Colour match VAS 100<br><br>6 months<br>C: 65.0±10.7<br>T: 77.0±9.5<br><br>12 months

N/R	N/R
N/R	N/R
C: 1.0±0.0<br>T:1.0±0.0	C: 1.0±0.2<br>T: 0.9±0.3

C: 0.8±0.4<br>T: 1.0±0.1	C: 0.9±0.3<br>T: 1.0±0.1
N/R	N/R
C: 1±0<br>T: 1±0.	C: 1±0<br>T: 1±0.
C: 1.0 ±0.0<br>T: 1.0±0.0	C: 0.91±0.3<br>T: 1.0±0.0
C: 0.87±0.34<br>T: 1.0	C: 0.92±0.97<br>T: 1.0±N/R

N/R	N/R
N/R	N/R
N/R	N/R

N/R	N/R
C: 0.81±0.4<br>T: 0.86±0.36	C: 0.88±0.34<br>T: 0.79±0.42
C: 1.0±0.0<br>T: 1.0±0.0	C: 0.73±0.46<br>T: 0.6±0.5

N/R	N/R
N/R	N/R

N/R	N/R
C: 0.9±0.1<br>T: 0.96±0.1	C: 1±0<br>T: 1±0

N/R	1= redder, 2= less red, 3= equally red<br><br>Baseline:<br>C: Redder: n=4, less red: n=0, equally red: n=26<br><br>T: C: Redder: n=8, less red: n=1, equally red: n=21<br><br>12 months<br>C: Redder: n=7, less red: n=0, equally red: n=23<br><br>T: Redder: n=5, less red: n=1, equally red: n=24
C: 0.9±0.2<br>T1: 0.9±0.2<br>T2: 1±0	C: 0.9±0.2<br>T1: 1±0<br>T2: 1±0

N/R	N/R
N/R	N/R
N/R	N/R
N/R	N/R

N/R	N/R

Keloids	Restoration/cervical lesion colour (0=not match, 3= good colour)
Excellent results for absence of keloid<br>C: 90.9 (n=20)<br>T: 95.45 (n=21)	N/R
Present/absent<br>C: absent n=15<br>T: absent n=16	N/R

N/R	N/R
N/R	N/R

N/R	N/R
N/R	N/R

N/R	N/R
N/R	N/R
N/R	N/R

N/R	N/R
N/R	N/R
N/R	N/R

N/R	N/R
N/R	N/R
N/R	N/R
N/R	N/R
N/R	N/R

Keloids (present)<br>C: 24% (n=6)<br>T: 12% (n=3)	N/R
N/R	N/R

N/R	N/R
N/R	N/R
N/R	N/R
N/R	N/R

Presence of keloids 1 year<br>C: 47%<br>T: 20%	N/R
Presence of keloids 1 year<br>C: 68%<br>T: 20%	N/R
N/R	N/R

N/R	N/R
N/R	N/R
N/R	C: 2.66±0.9<br>T: 2.82±0.9

N/R	N/R
Present/absent (yes)<br><br>6 months<br>C: 40% (n=10)<br>T: 16% (n=4)	N/R

N/R	N/R
N/R	N/R
N/R	N/R

N/R	N/R
N/R	N/R
N/R	C: 2.25±1.3<br>T: 2.45±0.6
N/R	N/R
N/R	N/R

N/R	N/R
N/R	N/R
N/R	N/R

N/R	N/R
N/R	N/R
N/R	N/R

N/R	N/R
N/R	N/R

N/R	N/R
N/R	C: 2.4±1.2<br>T: 2.7±0.9

N/R	N/R
N/R	N/R

N/R	N/R
N/R	N/R
N/R	N/R
N/R	N/R

N/R	N/R

RELE[illegible]	
Overall satisfaction	Aesthetic satisfaction
	4 point scale<br><br>C: Excellent: n=9, Good: n=11, Fair: n=1, Poor: n=1<br>T: Excellent: n=13, Good: n=9, Fair: n=0, Poor: n=0<br><br>Excellent results for amount of root coverage<br>C: 36.37 (n=8)<br>T: 59.09 (n=13)
Overall satisfaction<br>C: excellent n=7, good n=6, fair n=2, no response n=1<br>T: excellent n=9, good n=4, fair n=3, no response n=0	Colour match<br>C: excellent n=9, good n=5, fair n=1, no response n=1<br>T: excellent n=6, good n=8, fair n=2, no response n=0<br><br>Amount of coverage<br>C: excellent n=10, good n=4, fair n=0, no response n=2<br>T: excellent n=7, good n=6, fair n=2, no

N/R	4 point scale<br>(bad, sufficient, good, optimum)<br>C: 2.5±0.63<br>T: 3.5±0.51<br><br>80% (n=12) preferred the test site in terms of aesthetic
N/R	N/R

N/R	4 point scale<br>(bad, sufficient, good, excellent)<br>C: good n=5, excellent n=12<br>T: sufficient n=1, good n=7, excellent n=9<br><br>Which is the preferable technique?<br>n=4 patients SCTG<br>n=4 patients SCPF<br>n=9 patients with no differences
N/R	4 point scale<br>(bad, sufficient, good, excellent)<br>C: good n=5, excellent n=12<br>T: bad n=3, good n=6, excellent n=8<br><br>Which is the preferable technique?<br>n=7 patients SCTG<br>n=0 patients SCPF<br>n=10 patients with no differences

N/R	N/R
Patient preference<br>20% (5) preferred C<br>60% (15) preferred T<br>20% (5) no difference	N/R
N/R	Position of the gingival margin (VAS 0-100)<br>C: 0.7±1.1<br>T: 0.9±1.0

N/R	N/R
N/R	N/R
N/R	N/R

N/R	N/R
N/R	Six-Months Evaluation<br><br>Five point scale<br><br>C: Very Unsatisfied 5.9% (n=1), Unsatisfied 5.9% (n=1), Neutral 5.9% (n=1), Satisfied 17.6% (n=3), Very satisfied 64.7% (n=11)<br>T: Very Unsatisfied 0% (n=0), Unsatisfied 5.9% (n=1), Neutral 0% (n=0), Satisfied 17.6% (n=3), Very satisfied 76.5% (n=13)
N/R	Five point scale<br><br>C: Very Unsatisfied 0% (n=0), Unsatisfied 5.9% (n=1), Neutral 0% (n=0), Satisfied 35.3% (n=6), Very satisfied 58.8% (n=10)<br>T: Very Unsatisfied 0% (n=0), Unsatisfied 11.8% (n=2), Neutral 0% (n=0), Satisfied 35.3% (n=6), Very satisfied 52.9% (n=9)
Which of the surgery you would prefer?<br>n=8 microscope<br>n=6 no difference	Questionnaire<br>C: Excellent n=13, good, n=4, sufficient, n=3, bad n=4<br>T: Excellent n=22, good, n=2, sufficient, n=0, bad n=0
N/R	N/R

N/R	VAS (0=bad, average=50 and excellent=100)<br><br>C: 91.2±9.3<br>T: 89.6±7.9
N/R	Aesthetic satisfaction VAS 100<br>C: 75.0 ± 13<br>T: 80.4 ± 13.3

N/R	Aesthetic satisfaction VAS 100<br>C: 90±13<br>T: 92±10
N/R	Aesthetic satisfaction VAS 100<br>C: 87 ± 10.44<br>T: 95 ± 11.12
N/R	N/R
N/R	VAS (0 = very bad, 50 = average, 100 = excellent)<br><br>C: 77.0±9.0<br>T: 84.0±9.0

N/R	Root coverage VAS 100<br>C: 82.3±11.6<br>T: 84.3±9.7<br><br>Colour match<br>C:75.0±14.3<br>T: 86.3±11.3
N/R	Root coverage VAS 100<br>C: 9.04±1.06<br>T: 9.32±8.97<br><br>Colour match<br>C: 6.72±2.03<br>T: 8.16±1.86
N/R	VAS<br>Baseline<br>C: 4.31±1.60<br>T: 4.0±1.62<br><br>6 months<br>C: 8.61±1.75<br>T: 9.2±1.05

N/R	VAS<br>2 years<br>C: 9.70±0.46<br>T: 9.63±0.76
N/R	N/R
N/R	VAS scale<br>C: 8.29±2.23<br>T: 8.66±1.13

VAS<br>C: 8.38±2.46<br>T: 8.58±1.86	What is your esthetic concern about the recession?<br>C: Excessive tooth length 26.8% (n=11), Lack of gingiva 14.6% (n=6), Color contrast 0% (n=0), No complaints 56.1% (n=23)<br>T: Excessive tooth length 24.3% (n=10), Lack of gingiva 17.1% (n=7), Color contrast 0% (n=0), No complaints 56.1% (n=23)<br><br>Which method of treatment and esthetic outcome do you prefer?<br>Control 14.6% (n=6)<br>Test 14.6% (n=6)<br>No preference 70.8% (n=29)
N/R	VAS scale 100<br><br>6 months<br>C: 78.3±9.12<br>T: 83.6±10.7<br><br>12 months

N/R	VAS scale from 0 to 10, final score on how pleased they were with the overall esthetic results.<br>C: 9.62±0.57<br>T1: 9.38±0.92<br>T2: 8.63± 2.26<br>T3: 9.24±0.93
N/R	Aesthetic VAS 10<br>C: 8.04±1.1<br>T: 8.64±0.76
N/R	VAS 10<br>C: 9.0±2.4<br>T: 9.6±0.6

N/R	VAS 10<br>C: 8.9±4.6<br>T: 9.1±1.3
VAS Overall satisfaction (0= not satisfied 10=high satisfaction)<br>C: 8.20±1.6<br>T: 8.47±1.1	VAS aesthetic<br>(0= bad 10= good esthetic)<br>C: 8.20±1.6<br>T: 8.80±1.0
N/R	VAS 10<br>Baseline<br>C: 4.1±2.9<br>T: 4.6±2.3<br><br>6 months<br>C: 9.0±2.3<br>T: 9.1±2.2<br><br>12 months<br>C: 9.2±1.1
Patient satisfaction was assessed using a three-point rating scale: fully satisfied = 3; satisfied = 2; and unsatisfied = 1<br><br>C: 15.91±3.50<br>T: 18.63±1.91	N/R
N/R	N/R

N/R	VAS 10<br>C: 8.20±1.82<br>T: 9.47±0.92
N/R	N/R
N/R	Root coverage (VAS 10)<br>C: 9.5±0.9<br>T: 9.7±0.5

N/R	Patient esthetic satisfaction point scale<br><br>C: poor 5% (n=1), unsatisfied 0% (n=0), sufficient 15% (n=3), neutral 5% (n=1), satisfied 5% (n=1), as requested 20% (n=4), harmonic 10% (n=2), highly satisfied 20% (n=4) perfect 20% (n=4)<br><br>T: poor 5% (n=1), unsatisfied 5% (n=1), sufficient 10% (n=2), neutral 0% (n=0), satisfied 5% (n=1), as requested 25% (n=5), harmonic 10% (n=2), highly satisfied 20% (n=4) perfect 20% (n=4)
VAS (0 – 100)<br>C: 95.4±6.0<br>T: 90.9±10.7	VAS (0 – 100)<br>C: 91.2±9.8<br>T: 88.6±10.4
N/R	N/R

N/R	N/R
	4-point scale<br><br>C: Excellent: n=15, Good: n=5, Fair: n=0, Poor: n=0<br>T: Excellent: n=13, Good: n=6, Fair: n=2, Poor: n=1<br><br>Appearance (satisfactory or not)<br>C: 0.75±0.44<br>T: 0.50±0.51<br><br>Obtained RC (satisfactory or not)<br>C: 0.90±0.30<br>T: 0.54±0.51

	Position and satisfaction of the gingiva VAS<br>Baseline:<br>C: 0.68±1.70<br>T: 0.62±1.70<br>6 months:<br>C: 8.93±2.71<br>T: 9.50±1.41<br><br>Gingival colour<br>Baseline:<br>C: 0.68±1.88<br>T: 1.43±2.89<br>6 months:<br>C: 9.31±1.57<br>T: 9.37±1.25
N/R	VAS 10<br>C: 9.0±0.9<br>T: 9.2±1.1

Did you prefer C or T?<br>C: 53% (n=16)<br>T: 47% (n=14)	Did you prefer C or T?<br><br>C: 73% (n=22)<br>T: 27% (n=8)
N/R	VAS:<br>Baseline:<br>C: 3.4±2.8<br>T1: 4.8±2.6<br>T2: 5.1±3.0<br><br>6 months:<br>C: 9.0±1.6<br>T1: 9.4±1.1<br>T2: 9.3±1.8

N/R	4 point scale (1=bad, 2=optimum, 3=sufficient, 4=good)<br><br>6 months<br>C: 2.7±0.8<br>T: 3.7±0.6<br><br>12 months:<br>C: 2.5±0.8<br>T: 3.7±0.5
N/R	VAS 1-10<br>C: 9.00±1.41<br>T: 8.67±0.90
N/R	C: 2.92±2.27<br>T: 2.62±3.5
N/R	N/R

Which operated sites do you like most?<br>C: 50%<br>T: 50%	N/R

EVANT ENDPOINTS - Contr
Morbidity of the procedure/Reported discomfort
Excellent results for pain experience<br><br>C: 22.73 (n=5)<br>T: 31.81 (n=7)
N/R

4 point scale<br>(bad, bothersome, good, optimum)<br>C: 2.6±0.51<br>T: 3.1±0.35<br><br>46.6% (n=7) higher discomfort in control sites due to palatal wound<br>53.4% (n=8) no difference
Discomfort at 1 month<br>45% in the control group reported high discomfort<br>5% in the test group reported high discomfort<br>50% reported no differences between treatments<br><br>Discomfort at 3 months<br>25% in the control group reported discomfort

Pain as 4 point likert scale<br>(none, low, moderate, severe)<br>Day 1<br>C: 1.44<br>T: 0.00<br><br>Day 2<br>C: 0.24<br>T: 0.00<br><br>Day 3<br>C. 0.18<br>T: 0.00<br><br>Pain (VAS 100mm)<br>C: 30mm<br>T: 0mm<br><br>7 patients indicated greater discomfort for the SCTG due to the palate
N/R

N/R
N/R
Pain during the procedure (VAS 100)<br>C: 1.9±7.4<br>T: 3.2±8.9<br><br>Hardship perception of the procedure (VAS 100)<br>C: 23.3±19.4<br>T: 31.4±24.6<br><br>Pain Post-operative (VAS 100)<br>C: 7.9±15.6<br>T: 13.3±20.3

N/R
N/R
VAS scale 0 to 10 (no sensitivity to extreme pain)<br>Above level 3 for both C and T

N/R
N/R
VAS pain 100-mm<br>C: 4.7<br>T: 4.7
N/R

Pain VAS (0=bad, average=50 and excellent=100)<br><br>C: 77.2±16.4<br>T: 93.2±8.0<br><br>Chewing ability VAS<br>(0=bad, average=50 and excellent=100)<br><br>C: 72.8±14.3<br>T: 88.8±8.3
Hardship perception of the procedure VAS 100<br>C: 24.3±8.1<br>T: 41.3±20.0<br><br>Pain during surgery VAS 100<br>C: 2.9±6.1<br>T: 2.3±5.0<br><br>Pain 1 week (yes)<br>C: 36% (n=5)<br>T: 60% (n=9)<br><br>Pain 1 week VAS 100<br>C: 10.0±15.7<br>T: 19.3±20.9<br><br>Days of discomfort<br>C: 0.0±0.0<br>T: 0.9±1.4

N/R
N/R
N/R
Hardship perception of the procedure VAS (0= easy to cope, 100 0 = difficult to cope)<br><br>C: 55.3 ± 16.0<br>T: 61.9 ± 17.2<br><br>Pain VAS (0 = very bad, 50 = average, 100 = excellent)<br><br>C: 42.7 ± 23.1<br>T: 46.9 ± 22.7

Post-operative discomfort<br>VAS 100<br>C: 44.7±23.9<br>T: 24.3±20.1<br><br>Post-operative bleeding VAS 100<br>C: 34.0±21.9<br>T: 20.7±18.2<br><br>Inability to chew VAS 100<br>C: 48.7±23.1<br>T: 28.3±23.1
Post-operative discomfort<br>VAS 100<br>C: 8.16±0.85<br>T: 8.04±0.93<br><br>Post-operative bleeding VAS 100<br>C: 8.88±0.83<br>T: 9.0±0.87
VAS scale<br>C: 1.68±2.13<br>T: 2.45±2.50

N/R
Discomfort 1 week (VAS 10cm)<br>C: 4.3±1.0<br>T: 2.2±0.7
N/R

Pain/discomfort at 7 days (VAS 10)<br>C: 2.04±1.82<br>T: 2.32±2.08<br><br>Pain/discomfort at 14 days (VAS 10)<br>C: 0.59±0.91<br>T: 0.68±1.21
N/R

N/R
Pain VAS 10<br>C: 3.56±1.12<br>T: 1.52±0.59
Post-operative pain VAS (0=no pain 10= extreme pain)<br>C: 2.9±2.8<br>T: 1.3±1.4

N/R
Post-operative discomfort (0=no 10= extreme discomfort)<br>C: 5.5±2.4<br>T: 2.3±2.5
N/R
N/R
N/R

N/R
Pain intensity 1 week (VAS 10)<br>Median<br>C: 1.0±2.0<br>T: 0.0±1.0<br><br>Discomfort 1 week (VAS 10)<br>Median<br>C: 2.0±2.0<br>T: 1.0±1.0
Discomfort (VAS 10)<br>C: 8.5±0.8<br>T: 8.1±0.8

Pain assessments at 1 week<br>C: no pain 0% (n=0), a little pain 40% (n=8), a little more pain 35% (n=7), even more pain 20% (n=4), a whole lot of pain 5% (n=1), worst pain 0%(n=0)<br><br>T: C: no pain 0% (n=0), a little pain 55% (n=9), a little more pain 25% (n=5), even more pain 20% (n=4), a whole lot of pain 0% (n=0), worst pain 0%(n=0)
General discomfort/pain post-operative VAS<br>C: 29.4±12.2<br>T: 24.5±11.1<br><br>Number of days of discomfort<br>C: 2.6±0.5<br>T: 1.4±0.6
N/R

Analysis as a parallel group:

VAS 100 24h

C: 37.1±20.8

T: 15.5±13.1

VAS 100 48h

C: 17±12.5

T: 4.5±8.

VAS 72h

C: 8±5.7

T: 1.4±3.2

Experience

C: 0.30±0.47

T: 0.72±0.45

VAS Intra-op:<br>C: 0.37±1.08<br>T: 2.06±3.88<br><br>1 week:<br>C: 1.37±2.33<br>T: 2.06±2.29<br><br>2 week:<br>C: 0.00±0.00<br>T: 0.50±1.31
N/R

N/R
VAS (7 days)<br><br>C: 2.6±2.4<br>T1: 3.2±2.5<br>T2: 3.8±2.8

N/R
N/R
C: 1.69±5.38<br>T: 1.31±2.92
C: 2.75±0.85<br>T1: 4.30±0.97<br>T2: 2.8±1

VAS vestibular
C: 28.33±18.50
T: 31.25±26.98

VAS palatal
C: 31.67±29.79
T: 46.67±32.57

rol Group vs. Experimenta

PATIENT-REPORTE

Number of painkillers
N/R
N/R

N/R

N/R

Number of painkillers<br>Day 1<br>C: 0.53<br>T: 0.00<br><br>Day 2<br>C: 0.06<br>T: 0.00<br><br>Day 3<br>C: 0.06<br>T: 0.00
N/R

N/R
N/R
Number of painkillers<br>C: 0.7±1.2<br>T: 1.3±2.5

N/R
N/R
N/R

N/R
N/R
N/R
n=10 patients in each group took only 1 pill
N/R

N/R
Number of painkillers<br>C: 2.1±0.8<br>T: 3.3±1.7

N/R
N/R
N/R
N/R

Mean ibuprofen assumption in mg<br>C: 2520±1155mg<br>T: 1100±1137mg
Mean ibuprofen assumption in mg<br>C: 2136±460mg<br>T: 2040±434mg
Number of pills<br>C: 2.26±2.92<br>T: 1.8±1.73

N/R
N/R
N/R

N/R
N/R

N/R
N/R
Number pills taken<br>C: 1.6±2.1<br>T: 0.8±1.0

N/R
N/R
N/R
N/R
N/R

N/R
N/R
Dosage of painkillers<br>C: 1193.5±459.6mg<br>T: 1058.8±320.1mg

N/R
Number of pills<br>C: 2.6±0.9<br>T: 4.0±0.8
N/R

N/R
N/R

N/R
N/R

N/R
C: 2.0±2.9<br>T1: 2.3±2.2<br>T2: 2.4±3.0

N/R
N/R
N/R
C: 2.45±1.6<br>T1: 3.70±1.66<br>T2: 2.55±1.39

N/R

al Group(s)

ED OUTCOME MEASURES (PROMs)

Dental hypersensitivity
N/R
N/R

N/R
Only 1 patients at test site reported DH

n=7 patients had dental hypersensitivity at baseline and no one at the final follow-up (data per group not available)
n=3 patients had dental hypersensitivity at 30 months

N/R
N/R
Dental hypersensitivity at 6 months<br>C: 12%<br>T: 12%

Dental Hypersensitivity at baseline<br>C: 46.2%<br>T: 25%<br><br>Dental Hypersensitivity at 108 months<br>C: 0%<br>T: 0%
N/R
N/R

N/R
N/R
N/R
C: n=11 patients at baseline and 3 at final follow-up suffer hypersensitivity<br>T: n=11 patients at baseline and no one at final follow-up suffers hypersensitivity
N/R

Post-op sensitivity VAS<br>(0=bad, average=50 and excellent=100)<br><br>C: 94.0±6.5<br>T: 87.6±8.3
Present<br>C: 23.5%<br>T: 29.4%

Present<br>C: 0%<br>T: 8%
Present<br>C: 0%<br>T: 9%
N/R
N/R

N/R
N/R
VAS scale (0 = without pain, 10 = extreme pain)<br><br>Present/absent (Yes)<br>Baseline C: 75%<br>T: 65%<br><br>6 months<br>C: 10%<br>T: 10%

N/R
N/R
Present/absent (Yes)<br>Baseline<br>C: 94.4%<br>T: 88%<br><br>6 months<br>C: 44.4%<br>T: 5.5%

N/R
N/R

VAS sensitivity (from 0 no pain to 10 severe pain)
Baseline
C: 2.09±1.77,
T1: 3.62±2.11
T2: 3.83±2.32
T3: 3.68±3.11

6 months
C: 0.57±1.22
T1: 0.94±1.20
T2: 1.41±1.8
T3: 1.37±2.01

Differences:
C: 1.52±2.15
T1: 2.68±2.4
T2: 2.42±2.93
T3: 2.31±3.7

Schiff cold air sensitivity scale (0 = patient does not respond to air stimulus; 1 = patient responds to air stimulus, but does not request discontinuation; 2 = patient responds to air stimulus and requests discontinuation or moves away from it; 3 = patient responds

---

N/R

VAS (0=no pain, 10=extreme pain)
Baseline
C: 2.9±2.7
T: 2.9±2.5

6 months
C: 0.0±0.3
T: 0.2±0.5

Present/absent (yes)
Baseline

VAS (0=no pain, 10=extreme pain)<br>Baseline<br>C: 2.9±2.7<br>T: 2.9±2.5<br><br>24 months<br>C: 0.1±0.3<br>T: 0.1±0.2
Present/absent (yes)<br>Baseline<br>C: 60%<br>T: 55%<br><br>12 months<br>C: 20%<br>T: 0%
VAS (0= no pain, 10= extreme pain)<br>Baseline<br>C: 5.0±3.1<br>T: 3.7±3.3<br><br>6 months<br>C: 1.3±2.0<br>T: 0.6±1.8<br><br>Present/absent (Yes)<br>Baseline
N/R
N/R

VAS<br>Baseline: C: 4.47±2.59<br>T: 3.87±3.36<br><br>12 months<br>C: 0±0<br>T: 0±0<br><br>SCHIFF scale<br>Baseline C: 1.27±0.7<br>T: 1.13±0.92<br><br>12 months
N/R
N/R

N/R
VAS (0 – 100)<br>Baseline<br>C: 24.9±28.7<br>T: 29.1±29.6<br><br>12 months<br>C: 3.6±7.3<br>T: 1.9±4.9<br><br>Present/absent (yes)<br>Baseline<br>C: 56%<br>T: 64%
N/R

N/R
VAS<br>Baseline:<br>C: 6.20±1.79<br>T: 5.54±1.59<br><br>12 months<br>C: 0.30±1.12<br>T: 2.50±3.37

VAS<br>Baseline:<br>C: 2.75±4.04<br>T: 2.68±3.02<br><br>6 months:<br>C: 0.75±1.77<br>T: 0.62±1.20
VAS 10<br>6 months<br>C: 1.1±1.8<br>T: 1.1 ± 2.3<br><br>Yes/No<br>Baseline<br>C: 74.2% (n=23)<br>T: 67.7% (n=21)<br><br>6 Months<br>C: 35.4% (n=11)<br>T: 32.2% (n=10)

Present/absent (Yes)<br>Baseline<br>C: n=5<br>T: n=4<br><br>12 months<br>C: n=1<br>T: n=1
VAS<br>Baseline:<br>C: 3.4±3.0<br>T1: 3.9±3.8<br>T2: 4.1±3.7<br><br>6 months:<br>C: 0.8±2.2<br>T1: 1.0±2.2<br>T2: 1.0±1.7<br><br>Present absent (Yes)<br>Baseline:<br>C: n=18<br>T1: n=16<br>T2: n=18<br><br>6 months:<br>C: n=3

N/R
N/R
C: 1.31±3.76<br>T: 1.84±2.22
N/R

N/R

Complications	Non-aesthetic concerns
N/R	N/R
Uneventful healing in all patients	N/R

Control group: 3 Necrosis at the palatal site	N/R
Pain, bleeding, swelling, bruising, sensitivity	N/R

Uneventful healing in all patients	N/R
N/R	N/R

No major complications	N/R
Uneventful healing in all patients	N/R
Uneventful healing in all patients	N/R

N/R	N/R
No serious adverse events<br>n=25 subjects (78.1%) experienced 75 adverse events (50% mild contusion, 40.6% face swelling)	N/R
Uneventful healing in all patients	N/R

N/R	N/R
Uneventful healing in all patients	N/R
N/R	N/R
Uneventful healing in all patients	N/R
Uneventful healing in all patients	N/R

Uneventful healing in all patients	N/R
N/R	N/R

N/R	N/R
N/R	N/R
n=2 graft exposures/infections<br>C: n=1<br>T: n=1<br>n=1 Paresthesia in Test group	N/R
Uneventful healing in all patients	N/R

Uneventful healing in all patients	N/R
Uneventful healing in all patients	N/R
Uneventful healing in all patients	N/R

N/R	N/R
N/R	N/R
Uneventful healing in all patients	N/R

Uneventful healing in all patients	N/R
Uneventful healing in all patients	N/R

N/R	N/R
N/R	N/R
Uneventful healing in all patients	N/R

N/R	N/R
N/R	N/R
Uneventful healing in all patients	N/R
Uneventful healing in all patients	N/R
N/R	N/R

N/R	N/R
N/R	N/R
Uneventful healing in all patients	N/R

N/R	N/R
No	N/R
N/R	N/R

Analysis as a parallel group<br>hematoma and swelling:<br>24h<br>C: 58.3%<br>T: 18.2%<br><br>48h<br>C: 58.3%<br>T: 18.2%<br><br>72h<br>C: 58.3%	N/R
N/R	N/R

Uneventful healing in all patients	NR
Uneventful healing in all patients	N/R

Uneventful healing in all patients	N/R
Uneventful healing in all patients	N/R

N/R	N/R
Uneventful healing in all patients	N/R
n=1 Necrosis of the graft in the SCTG group	N/R
N/R	N/R

None	N/R

Quality of life scores	CRC (%)
N/R	C: 50<br>T: 31.8
N/R	C:43.8<br>T: 43.8

N/R	C: 80<br>T: 86.7
N/R	C: 79<br>T: 89.5

N/R	C: 76.47<br>T: 52.94
N/R	C: 88.24<br>T: 58.82

N/R	C: 60<br>T: 33.3
N/R	N/R
Daily-life interference (VAS 100)<br>C: 24.4±22.8<br>T: 21.9±22.7<br><br>Job interference (VAS 100)<br>C: 6.2±14.2<br>T: 7.3±15.3<br><br>Relationship interference (VAS 100)<br>C: 15.9±22.8<br>T: 18.5±29.4	C: 37<br>T: 60

N/R	C: 38.5<br>T: 66.7
N/R	N/R
N/R	C: 83<br>T: 92

N/R	C: 60<br>T: 70
N/R	Six-Months Evaluation<br><br>C: 94.1<br>T: 70.6
N/R	C: 88.2<br>T: 52.9
N/R	C: 58.3<br>T: 87.5
N/R	C: 70<br>T: 80

N/R	C: 48<br>T: 4
N/R	C: 29<br>T: 57

N/R	C: 36<br>T: 69
N/R	C: 20<br>T: 63
N/R	N/R
N/R	C: 46.7<br>T: 81.2

N/R	C: 80<br>T: 83
N/R	C: 48<br>C: 88
N/R	C: 35<br>T: 65

N/R	C: 76<br>T: 79
OHIP-14 questionnaire<br>(difference 6 months-BL)<br>C: 26.1±9.4<br>T: 25.7±9.2	C: 80.8<br>T: 84.6
N/R	C: 16.6<br>T: 5.5

N/R	C: 38<br>T: 42
N/R	6months<br>C: 92<br>T: 84<br><br>12 months<br>C: 88<br>T: 80

OHIP-14 questionnaire<br>Baseline<br>C: 8.0±8.80<br>T1: 14.76±9.61<br>T2: 13.35±8.86<br>T3: 10.29±7.88<br><br>6 months<br>C: 5.12±6.58<br>T1: 3.47±4.72<br>T2: 4.71±4.83<br>T3: 6.88±9.99<br><br>Differences:<br>C: 2.88±10.98<br>T1: 11.29±10.71<br>T2: 8.64±10.09<br>T3: 3.41±12.72	C: 23.53<br>T1: 52.94<br>T2: 70.59<br>T3: 58.82
N/R	C: 68<br>T: 92
N/R	C: 71.4<br>T: 28.6

N/R	C: 68.4<br>T: 50
N/R	C: 35<br>T: 80
N/R	C: 70<br>T: 60
N/R	C: 45.45<br>T: 72.73
N/R	C: 52<br>T: 68

N/R	C: 33.33<br>T: 66.67
N/R	C: 33<br>T: 80
N/R	C: 80.6<br>T: 88.2

N/R	C: 80<br>T: 70
N/R	C: 50<br>T: 71
N/R	C: 90.9<br>T: 65

N/R	N/R
N/R	C: 60<br>T: 45.45

NR	C: 50<br>T: 37.5
N/R	C: 55.2±21.8<br>T: 54.4±20.0

N/R	N/R
N/R	C: 48<br>T1: 52<br>T2: 28

N/R	6 months<br>C: 87.5<br>T: 81.2<br><br>12 months<br>C: 43.7<br>T: 68.7
N/R	N/R
N/R	C: 54<br>T: 54
N/R	C: 73<br>T1: 83<br>T2: 74

N/R	C: 58.3<br>T: 41.7

CLINICAL OU	
MRC (%)	REC RED or final values (mm)
C: 74.1±38.3<br>T: 65.9±46.7	C: 2.2±1.1<br>T: 1.7±1.2
C: 84.0±25.0<br>T: 73.0±26.0	C: 2.8±0.8<br>T: 2.5±0.7

C: 94.7±11.21<br>T: 97.3±7.01	C: 3.6±0.72<br>T: 3.9±0.71
C: 93.8<br>T: 95.1	C: 4.01±0.4<br>T: 4.07±0.4

C: 96.10±7.69<br>T: 90.95±11.46	C: .2.05±0.52<br>T: 1.99±0.50
C: 96.83<br>T: 89.25	C: 2.08±0.62<br>T: 1.92±0.79

C: 84.81<br>T: 68.98	C: 2.32±0.90<br>T: 2.09±1.23
N/R	C: 0.38±0.28<br>T: 0.24±0.28
N/R	C: 1.5±1.1<br>T: 2.0±1.0

N/R	C: 1.4±1.3<br>T: 1.9±0.94
C: 98.6±4.16<br>T: 90.8±11.7	C: 3.3±0.6<br>T: 2.9±0.5
N/R	C: 2.05±0.64<br>T: 2.21±0.60

C: 83.2±22.22<br>T: 83.2±27.21	C: 2.3±0.8<br>T: 1.9±0.7
Six-Months follow-up<br><br>C: 97.0 ± 9.18<br>T: 83.5 ± 23.1<br><br>One-year follow-up<br><br>C: 99.3 ±22.19<br>T: 88.5 ±2.67	Six-Months follow-up<br><br>C: 3.10 ± 0.74<br>T: 2.62 ± 0.48<br><br>One-year follow-up<br>C: 3.17±0.32<br>T: 2.78±0.58
C: 95.5±12.8<br>T: 77.6±29.2	N/R
C: 88.3<br>T: 98.0	C: 2.24±0.64<br>T: 2.46±0.38
C: 88.5±19.4<br>T: 92.6±15.7	C: 2.7±0.11<br>T: 3.3±0.17

C: 88.8±11.2<br>T: 74.2±8.2	C: 4.04±1.02<br>T: 3.68±0.75
C: 69.0±20.1<br>T: 85.0±17.2	C: 2.0±0.7<br>T: 2.6±0.7

C: 69.7±26.7<br>T: 86.5±69.7	C: 2.4±0.9<br>T: 2.8±0.9
C: 55.5±24.8<br>T: 87.1±18.3	C: 2.2±0.8<br>T: 2.7±0.8
C: 77.21±29.10<br>T: 71.01±32.87	C: 2.06±1.11<br>T: 2.0±0.87
C: 75.7±24.2<br>T: 92.5±16.1	C: 3.5±0.9<br>T: 4.8±0.8

N/R	C: 3.66±0.96<br>T: 3.80±0.92
N/R	C: 3.08±1.12<br>T: 3.68±1.11
C: 89.38±22.3<br>T: 91.84±22.5	C: 2.74±0.74<br>T: 2.85±0.95

C: 92.32±15.0<br>T: 93.43±14.3	C: 3.07±0.66<br>T: 2.88±0.78
C: 95.9<br>T: 96.3	C: 4.0±1.2<br>T: 4.4.±1.1
C: 82.16±16.1<br>T: 73.84±19.2	C: 2.82±0.74<br>T: 2.72±0.69

C: 75.05±26.24<br>T: 76.28±28.07	C: 2.26±1.17<br>T: 2.48±1.46
N/R	C: 3.17±0.89<br>T: 2.93±1.01

C: 68.04±24.11<br>T1: 87.20±15.01<br>T2: 88.77±20.66<br>T3: 91.59±11.08	C: 2.16±0.75<br>T1: 2.71±0.51<br>T2: 2.67±0.57<br>T3: 2.91±0.53
C: 90.48±15.06<br>T: 97.64±8.27	C: 3.88±1.08<br>T: 4.32±1.16
C: 87.2±27.1<br>T: 77.4±20.4	C: 2.8±1.0<br>T: 2.4±0.9

C: 89.5±14.6<br>T: 87.7±18.4	C: 2.8±0.6<br>T: 2.7±0.9
C: 72.5±22.4<br>T: 92.3±16.6	C: 1.57±1.02<br>T: 2.24±1.14
C: 92.2±28.4<br>T: 93.0±26.1	C: 2.4±1.1<br>T: 2.5±1.0
C: 77.25±25.78<br>T: 94.80±9.60	C: 3.77±0.61<br>T: 3.04±1.06
N/R	C: 2.13±2.12<br>T: 2.57±1.63

C: 79.76±17.44<br>T: 90.86±14.69	C: 1.98±0.33<br>T: 1.85±0.41
C: 73.1±20.8<br>T: 93.8±13.0	Median and IQR<br>C: 1.9±1.0<br>T: 2.7±1.0
C: 94.6±11.9<br>T: 97.1±8.3	C: 3.6±1.22<br>T: 3.8±1.24

C: 96.48±7.49<br>T: 94.22±10.99	C: 2.42±0.52<br>T: 2.47±0.47
N/R	C: 2.7±0.6<br>T: 3.1±0.7
C: 79.1<br>T: 81.3	C: 2.73±0.67<br>T: 2.6±0.5

Analysis as a parallel group:<br>C: 97.6±5.7<br>T: 74.2±18.8<br><br>Analysis as a split-mouth group:<br>C: 97.1±6.2<br>T: 75.0±19.6	Analysis as a parallel group:<br>C: 3.2±0.7<br>T: 2.4±0.7<br><br>Analysis as a split-mouth group:<br>C: 3.1±0.8<br>T: 2.4±0.7
C: 85.60±30.47<br>T: 35.60±13.91	C: 1.33 ±0.86<br>T: 0.76 ±0.82

C: 76.4±30.2<br>T: 68.2±33	C: 2.29±1.24<br>T: 2.03±1.26
C: 70.3±22<br>T: 69±21.6	C: 1.9±0.8<br>T: 2.0±0.7

C: 84.49±19.98<br>T: 63.2±31.56	C: 3.11±1.13<br>T: 2.26±1.58
C: 78.95±26.2<br>T1: 78.0±28.5<br>T2: 65.6±26.9	C: 2.4±0.8<br>T1: 2.4±0.9<br>T2: 2.1±0.8

6 months<br>C: 95.9±12.8<br>T: 92.8±13.7<br><br>12 months<br>C: 90.0±16.2<br>T: 90.7±14.0	C: 3.1±1.2<br>T: 3.6±1.5
C: 88.66±33.18<br>T: 69.2±22.91	C: 2.6±1.08<br>T: 1.66±1.11
N/R	C: 1.69±2.62<br>T: 1.38±1.19
C: 75<br>T1: 86<br>T2: 80.15	C: 3.05±1.10<br>T1: 3.70±0.73<br>T2: 3.28±0.59

C: 80.36±29.66<br>T: 69.43±34.79	C: 1.53±0.66<br>T: 1.44±0.89

UTCOMES

Changes in KTW or final values (mm)	Changes in GT or final values (mm)
C: 1.6±1.9<br>T: 1.2±1.3	N/R
C: 1.1±2.0<br>T: 0.7±1.7	N/R

C: 3.3±0.72<br>T: 2.3± 0.59	N/R
C: 1.56±0.44<br>T: 0.71±0.46	N/R

C: 1.15±0.71<br>T: 0.90±0.83	C: 0.46±0.44<br>T: 0.03±0.32
C: 1.14±1.46<br>T: 0.86±1.77	C: 0.33±0.38<br>T: 0.07±0.33

C: 0.14±1.28<br>T: 0.32±0.81	C: 0.51±0.31<br>T: 0.66±0.44
C: 3.29±0.68<br>T: 1.33±0.68	N/R
C: -0.1±1.2<br>T: 0.6±1.1	N/R

C: -0.2±1.66<br>T: 1.6±1.22	N/R
C: 1.3±0.41<br>T: 1.0±0.41	N/R
C: 0.09±1.69<br>T: 0.69±1.15	C: -0.04±0.65<br>T: 0.31±0.5

C: 0.8±0.78<br>T: 0.5±1.18	N/R
Six-Months follow-up<br><br>C: 1.26±1.07<br>T: 1.34±0.71<br><br>One-year follow-up<br>C: 1.09±1.68<br>T: 1.11±0.87	N/R
N/R	N/R
C: 1.37±1.18<br>T 1.51±1.01	C: 0.34±0.39<br>T: 0.30±0.36
C: 0.3±0.11<br>T: 0.3±0.05	N/R

C: 0.96±0.73<br>T: 2.08±0.91	C: 0.80±0.13<br>T: 0.43±0.27
C: 0.1±1.13<br>T: 0.2±1.08	N/R

C: 0.0±0.8<br>T: 1.92±1.18	N/R
C: 0.5±1.2<br>T: 2.1±1.4	N/R
C: 0.55±1.45<br>T: 0.72±1.07	1mm apical to the GM<br>C: 0.50±0.69<br>T: 0.32±0.70<br><br>3mm apical to the GM<br>C: 0.99±0.83<br>T: 0.66±0.90
C: 4.9±0.7<br>T: 4.0±1.2	N/R

C: 2.17±0.59<br>T: 2.50±0.73	C: 0.72±0.12<br>T: 1.39±0.14
C: 2.2±1.77<br>T: 1.56±1.10	C: 0.58±0.46<br>T 0.78±0.66
C: 0.69±1.32<br>T: 0.41±1.57	C: 0.83±1.09<br>T: 0.67±0.53

C: 0.74±1.34<br>T: 0.01±1.27	C: 0.84±0.30<br>T: 0.61±0.30
C: 4.3±1.1<br>T: 4.6±1.3	N/R
C: 0.58 ±1.2<br>T: 0.45±0.7	C: 0.92±0.3<br>T: 1.07±0.3

C: 0.64±1.05<br>T: 1.06±1.07	C: 0.27±0.43<br>T: 0.52±0.46
C: 0.6±0.78<br>T: 0.51±0.65	N/R

C: 0.30±1.4<br>T1: 0.35±1.04<br>T2: 0.36±0.9<br>T3: 0.34±0.86	C: 0.13±0.32<br>T1: 0.36±0.47<br>T2: 0.12±0.27<br>T3: 0.30±0.31
C: 3.88±1.27<br>T: 4.36±1.29	N/R
C: 1.0±0.9<br>T: 0.7±0.45	C: 1.0±0.4<br>T: 0.7±0.45

C: 1.1±1.2<br>T: 1.0±1.2	C: 1.0±0.4<br>T: 0.7±0.5
C: 0.6±1.28<br>T: 0.2±0.7	N/R
C: 1.2±1.0<br>T: 1.4±1.3	C: 1±0.5<br>T: 1±0.5
C: 4.82±0.98<br>T: 2.82±0.64	C: 0.69±0.09<br>T: 0.06±0.05
N/R	N/R

C: 0.19±0.57<br>T: 0.10±0.35	C: 0.36±0.27<br>T: 0.35±0.28
Median and IQR<br>C: 0.0<br>T: 0.0	N/R
C: 2±1.7<br>T: 2.8±1.77	C: 0.7±1.17<br>T: 0.9±1.12

C: 1.30±0.44<br>T: 1.35±0.40	N/R
C: 0.2 ± 0.7<br>T: 1.7 ± 0.7	C: 0.06±0.18<br>T: 0.6±0.15
N/R	N/R

Analysis as a parallel group:<br>C: 1.2±1.9<br>T: 1.6±1.7<br><br>Analysis as a split-mouth group:<br>C: 0.6±1.7<br>T: 1.8±1.4	Analysis as a parallel group:<br>C: 0.2±0.8<br>T: -0.1±1.0<br><br>Analysis as a split-mouth group:<br>C: 0.0±0.7<br>T: -0.2±0.8
C: 1.70 ±1.49<br>T: 0.36 ±0.96	C: 0.8 ±0.53<br>T: 0.52 ±0.59

C: 1.37±1.10<br>T: 5.40±1.80	N/R
C: 0.4±0.7<br>T: 0.9±0.8	C: 0.1±0.3<br>T: 0.7±0.2

C: 1.3±0.88<br>T: 0.8±1.8	C: 86.02 ± 82.4 mm3<br>T: 150.2 ± 80.1 mm3<br><br>C: Thin: 0, Thick: 30<br>T: Thin: 3, Thick: 27
C: 0.4±1.1<br>T1: 0.2±1.0<br>T2: 0.1±0.7	C: 0.0±0.1<br>T1: 0.4±0.3<br>T2: 0.4±0.2

C: 1.4±1.8<br>T: 1.5±1.6	C: 1.6±0.6<br>T: 1.1±0.2
C: 0.8±1.01<br>T: 0.47±0.82	N/R
C: 1.66±1.63<br>T: 1.27±2.04	N/R
C: -0.15±0.67<br>T1: 2.0±0.97<br>T2: 0.08±0.25	C: 0.31±0.10<br>T1: 0.99±0.02<br>T2: 0.92±0.52

N/R	C: 1.01±0.35<br>T: 1.07±0.33

Wound Healing Index
N/R
N/R

Flap dehiscence (from the CEJ to the margin of the flap at 2 weeks)<br>C: 53.4%<br>T: 13.3%
N/R

N/R
N/R

N/R
N/R
N/R

N/R
N/R
N/R

N/R
N/R
N/R
N/R
N/R

N/R
N/R

N/R
N/R
N/R
N/R

Early (2 weeks) shrinkage of the covering flap<br>C: 26.6%<br>T: 6.6%<br><br>Graft exposure at 1 year<br>C: 53%<br>T: 17%
Early (2 weeks) shrinkage of the covering flap<br>C: 36%<br>T: 4%<br><br>Graft exposure at 1 year<br>C: 60%<br>T: 20%
N/R

N/R
N/R
N/R

N/R
N/R

N/R
N/R
N/R

N/R
N/R
N/R
N/R
N/R

N/R
N/R
N/R

N/R
N/R
N/R

N/R
2 weeks healing (Uneventful= 1<br>Slightly disturbed=2<br>Poor=3)<br>C: 1.65±0.67<br>T: 1.95±0.89

N/R
N/R

N/R
N/R

N/R
N/R
N/R
N/R

N/R

Acronyms

ADM (Acellular Dermal Matrix)
A-PRF (Advanced-platelet Rich Fibrin)
b-TCP/rhPDGF-BB (Beta-tricalcium Phosphate/Recombinant Human Platelet-derived Growth Factor-BB)
BCT (Bilayered Cell Therapy)
CAF (Coronally Advanced Flap)
CEJ (Cemento-enamel Junction)
CM (Collagen Matrix)
CMX (Xenogeneic Collagen Matrix)
CRC (Complete Root Coverage)
EMD (Enamel Matrix Derivative)

EMD (Enamel Matrix Derivative)

FDADM (Freeze-dried Acellular Dermal Matrix)

FGG (Free Gingival Graft)

GM (Gingival Margin)

GT (Gingival Thickness)

GTR (Guided Tissue Regeneration)

GUG (Gingival Unit Graft)

HA (Hyaluronic Acid)

iPRF (Injectable Platelet Rich Fibrin)

KTW (Keratinized Tissue Width)

LMCAF (Laterally Moved Coronally Advanced fFap)

LALPF (Laser-assisted Laterally Positioned Flap)

LLLT (Low Level Laser Therapy)

LPF (laterally Positioned Flap)

L-PRF (Leucocyte Platelet Rich Fibrin)

LST (Labial Submucosal Tissue)

MCAF (Modified CAF)

MGJ (Mucogingival Junction)

MRC (Mean Root Coverage)

MRES (Modified Recession Esthetic Score)

NCCL (Non-carious Cervical Lesion)

PCM (Porcine Collagen Matrix)

PPF (Periosteal Pedicle Flap)

PPF (Periosteal Pedicle Flap)
QCE (Qualitative Cosmetic Evaluation)
REC (Gingival Recession)
RecRED (Recession Reduction)
RES (Recession Esthetic Score)
SCPF (Semilinar Coronally Positioned Flap)
SCTG (Sub-epithelial Connective Tissue Graft)
SDADM (Solvent-dehydrated Acellular Dermal Matrix)
VCMX (Cross-linked Volume Stable Collagen Matrix)
XDM (Xenogeneic Acellular Dermal Matrix)
