# Supplementary material for: Clinician‐ and patient‐reported outcomes following the surgical treatment of single gingival recession defects: A systematic review
Source: Periodontol 2000. 2025 Jul 22;99(1):7–20. doi: 10.1111/prd.12641 (PMC13428094; doi:10.1111/prd.12641)
Supplement: Supplementary file 3 — Table S1 [file PRD-99-7-s003.docx]

#1 gingival recession
#2 recession near gingiva* OR recession near defect* OR recession‐type defect*
#3 exposure near root* OR exposed near root*
#4 gingiva* near defect*
#5 denude* near root surface*
#6 #1 OR #2 OR #3 OR #4 OR #5
#7 Guided tissue regeneration
#8 tissue near regenerat*
#9 gingiva* near esthetic* OR gingiva* near aesthetic*
#10 periodont* and plastic surgery
#11 soft tissue graft OR coronally advanced flap
#12 laterally positioned flap* OR laterally‐positioned flap
#13 connective tissue graft* OR connective‐tissue graft*
#14 gingiva* near transplant*
#15 dermal matrix near graft*
#16 enamel matrix protein OR biologics OR autologous blood products
#17 #7 OR #8 OR #9 OR #10 OR #11 OR #12 OR #13 OR #14 OR #15 OR #16
#18 #6 AND #17

**Table S1.** Search strategies used in each of the three databases. Search strategies in CENTRAL and EMBASE were modeled based on the search strategy designed for PubMed using the filters: humans and date of publication from 1/1/2000 - 3/31/2024.
