# Supplementary material for: Clinician‐ and patient‐reported outcomes following the surgical treatment of single gingival recession defects: A systematic review
Source: Periodontol 2000. 2025 Jul 22;99(1):7–20. doi: 10.1111/prd.12641 (PMC13428094; doi:10.1111/prd.12641)
Supplement: Supplementary file 4 — Table S2 [file PRD-99-7-s001.docx]

| **Articles excluded** | **Reason for exclusion** |
| --- | --- |
| 2023 Zeytinci et al | Insufficient sample size |
| 2023 Tavelli et al. | Inclusion of multiple gingival recession defects |
| 2023 Naziker et al. | Inclusion of multiple gingival recession defects |
| 2023 Bommala et al. | Inclusion of multiple gingival recession defects |
| 2023 Anegundi et al. | Missing primary outcomes of interest |
| 2022 Savithri et al. | Inclusion of multiple gingival recession defects |
| 2022 Quispe-Lopez et al. | Insufficient sample size |
| 2022 Miguel et al. | Non-RCT |
| 2022 Mashaly et al. | Inclusion of multiple gingival recession defects |
| 2022 Ezz Elarab et al. | Poster presentation |
| 2022 Barbato et al. | Poster presentation |
| 2022 Amer et al. | Poster presentation |
| 2022 Zhan et al. | Non-RCT and Inclusion of multiple gingival recession defects |
| 2021 Srivastava et al. | Discrepancies in methods and reported outcomes |
| 2021 Zuhr et al. | Inclusion of multiple gingival recession defects |
| 2021 Helal et al. | Poster presentation |
| 2021 Garzon et al. | Insufficient sample size |
| 2020 Suzuki et al. | Inclusion of multiple gingival recession defects |
| 2020 Salem et al. | Inclusion of multiple gingival recession defects |
| 2020 Muthuraj et al. | Inclusion of multiple gingival recession defects |
| 2019 Pilloni et al. | Inclusion of multiple gingival recession defects |
| 2019 de Resende et al. | Surgical treatment not aimed at root coverage |
| 2019 Barootchi et al. | Inclusion of multiple gingival recession defects |
| 2018 Culhaoglu et al. | Inclusion of multiple gingival recession defects |
| 2016 Jenabian et al. | Insufficient sample size |
| 2016 Gobbato et al. | Inclusion of multiple gingival recession defects |
| 2016 Azaripour et al. | Inclusion of multiple gingival recession defects |
| 2015 Nizam et al. | Inclusion of multiple gingival recession defects |
| 2015 Jindal et al. | Inclusion of multiple gingival recession defects |
| 2014 Zuhr et al. | Inclusion of multiple gingival recession defects |
| 2014 Salhi et al. | Inclusion of multiple gingival recession defects |
| 2014 Moka et al. | Missing primary outcomes of interest |
| 2014 Bajic et al. | Other than English language |
| 2010 Zucchelli et al. | Inclusion of multiple gingival recession defects |
| 2007 Joly et al. | Missing primary outcomes of interest |
| 2007 Mahajan et al. | Insufficient sample size |
| 2004 Cheung et al. | Inclusion of multiple gingival recession defects |

**Table S2.** List of excluded articles and the reasons for exclusion.
